# Collagen-elastin dermal scaffolds enhance tissue regeneration and reduce scarring in preclinical models

**DOI:** 10.1016/j.mtbio.2025.102239

**Published:** 2025-08-25

**Authors:** Roman Krymchenko, Nancy Avila-Martinez, Merel Gansevoort, Gert-Jan Bakker, Madalena L.N.P. Gomes, Marcel Vlig, Elly M.M. Versteeg, Bouke K.H.L. Boekema, Toin H. van Kuppevelt, Willeke F. Daamen

**Affiliations:** a Radboud University Medical Center, Research Institute for Medical Innovation, Department of Medical BioSciences, Geert Grooteplein 28, 6525 GA, Nijmegen, the Netherlands; b Alliance of Dutch Burn Care, Burn Research Lab, Zeestraat 29, 1941 AJ, Beverwijk, the Netherlands; c Department of Pathology, Amsterdam University Medical Center (AUMC), Meibergdreef 9, 1105 AZ, Amsterdam, the Netherlands; d Tissue Function and Regeneration, Amsterdam Movement Sciences Research Institute, Meibergdreef 9, 1105 AZ, Amsterdam, the Netherlands; e Department of Plastic, Reconstructive and Hand Surgery, AUMC, Location VUmc, De Boelelaan 1117, 1081 HV, Amsterdam, the Netherlands

**Keywords:** Biomaterials, Elastic fibers, Rat model, Skin healing, In vivo, In vitro

## Abstract

Severe scarring is an inevitable consequence of large full-thickness skin wounds, often leading to long-term complications that affect patients’ well-being and necessitate extended medical interventions. While autologous split-thickness skin grafts remain the clinical standard for wound treatment, they frequently result in contractures, excessive scarring, and the need for additional corrective procedures. To address these challenges, bioengineered skin substitutes capable of promoting efficient healing while reducing complications are highly desirable. Elastin, an essential component of the extracellular matrix, plays a crucial role in restoring tissue elasticity and regulating scar formation during wound healing. This study explores the impact of two distinct elastin-derived components, produced through acidic and basic hydrolysis, on wound repair. We developed and characterized collagen-based scaffolds enriched with these elastin hydrolysates and assessed their influence on different types of human skin fibroblasts, including fetal, eschar-derived, and healthy adult dermis-derived fibroblasts. Furthermore, we evaluated their therapeutic potential in a preclinical rat model. Our findings indicated that fetal fibroblasts exhibited the most pronounced extracellular matrix deposition and cellular infiltration within the scaffolds, followed by eschar fibroblasts and, lastly, healthy adult cells. The incorporation of elastin into collagen scaffolds led to a reduction in α-SMA protein expression, a biomarker of fibrosis, compared to collagen-only scaffolds. Notably, collagen scaffolds supplemented with elastin hydrolysate from basic hydrolysis demonstrated the most promising outcomes for scarless healing, characterized by minimal wound contraction, enhanced extracellular matrix formation, and increased neovascularization.

## Highlights

• Elastins solubilized by alkaline or acidic hydrolysis were incorporated in collagen scaffolds.
• Fetal fibroblasts showed strongest ECM deposition and infiltration into scaffolds.
• Supplementation of collagen scaffolds with elastin reduced α-SMA expression *in vitro*.
• Scaffolds with elastin from alkaline hydrolysis favored scarless healing *in vivo*.

## Graphical abstract

**Figure:**
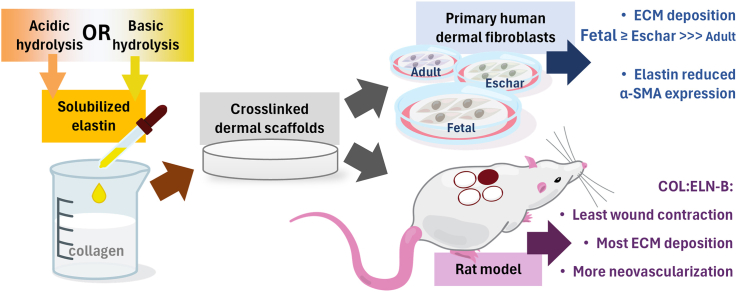

## 1 Introduction

Skin wounds compromise the body's primary barrier and can vary from minor abrasions to deep tissue damage caused by explosions, trauma, surgeries, burns, or ischemia. Healing occurs in four overlapping stages: hemostasis, inflammation, proliferation, and remodeling [1]. While minor wounds generally heal easily, severe deep wounds take longer to heal due to the destruction of essential stem cell populations on the basement membrane and lack of skin appendages [2]. In contrast to scarless healing observed in fetal skin [3], full-thickness skin wounds do not regenerate fully in adults, leading to severe scarring that impacts patients' quality of life and often requires prolonged treatment [4].

Although dry necrotic tissue (eschar) may temporarily cover a wound, it can impede healing and mask underlying processes, posing additional risks [5]. Therefore, removal of the eschar tissue is a critical step, as it allows for the assessment of the wound bed and enables the formation of a well-vascularized dermis, which is an essential prerequisite for successful re-epithelialization and wound closure [6]. The standard of care method, autologous split-thickness skin grafts, involves harvesting healthy skin (primarily the epidermis and a small part of the dermis) and applying it to the injured area. However, this procedure still leads to significant scarring, contractures, and complications, which often require follow-up treatments such as scar revision surgery [7]. Therefore, appropriate bioengineered dermal scaffolds are necessary to minimize the complications while supporting and accelerating healing [8,9].

Animal-derived collagen is a versatile and effective biomaterial in a range of applications, especially in wound healing [10]. Collagen, the main component of dermis, provides a suitable environment for fibroblasts, promoting tissue regeneration and accelerating the healing process [11]. Collagen porous scaffolds effectively absorb wound exudate, maintain a moist environment, and reduce the risk of infection and mechanical damage [12]. Their porous structure facilitates nutrient and cell transport, enhancing cell adhesion, proliferation, and migration. When applied to severe wounds, these biomaterials serve as frameworks for new tissue formation, aiding soft tissue repair and improving recovery outcomes.

In contrast to collagen, another key extracellular matrix (ECM) protein, elastin, is often neglected despite its vital role in maintaining skin elasticity and resilience [13]. In adults, elastic fibers regenerate slowly after injury and are significantly reduced in scar tissue [14]. Soluble elastins have been reported to show various beneficial effects crucial for wound healing, including the stimulation of cell adhesion and proliferation, induced angiogenesis, and boosted elastin expression [[15], [16], [17], [18], [19], [20], [21]]. Based on this, different porous type I collagen scaffolds supplemented with solubilized elastin have been developed [[22], [23], [24], [25], [26], [27], [28], [29]]. Some studies have reported the mechanical characterization of these collagen-elastin scaffolds [29] and the effects of crosslinking [25,30]. More importantly, the supplementation of elastin in such scaffolds has demonstrated a higher potential for skin regeneration compared to collagen-only analogues [22,23,25,26]. However, despite these promising observations and the established bioactivity of soluble elastin, a comprehensive understanding of its precise role in the complex cascade of wound healing and regeneration remains limited. This is partly attributed to the inherent complexity of the wound microenvironment, also to the limited investigation into how different chemical solubilization methods affect the molecular properties and biological activity of soluble elastin, and thereby its therapeutic potential. A complete elucidation of the mechanisms within the complex regenerative cascade is, however, beyond the scope of this study.

In this study, we investigate and directly compare the effects of two types of chemically solubilized elastins, obtained through acidic or alkaline hydrolysis, on wound healing. We created and characterized novel collagen-elastin scaffolds and evaluated their effects on various types of fibroblasts, including normal adult, eschar, and fetal fibroblasts. Additionally, we examined the regenerative and scarring outcomes of these scaffolds in a rat model, providing the first side-by-side comparison.

## 2 Materials and methods

Unless otherwise specified, chemicals, enzymes, and cell culture supplements were purchased from Sigma-Aldrich/Merck, while cell culture media and antibiotics were obtained from Gibco. Elastin fibers were isolated from equine *ligamentum nuchae* and purified using a protocol established in our laboratory [31].

### 2.1 Preparation and characterization of solubilized elastin peptides

#### 2.1.1 Solubilized elastin by acidic hydrolysis (ELN-A)

4.1 g of dry elastin fibers were dispersed in 90 mL of 0.25 M oxalic acid and maintained at 95 °C for 9 h, ensuring complete solubilization. Following incubation, the solution underwent centrifugation at 500×*g* for 5 min to eliminate residual contaminants. The resulting elastin solution was then adjusted to approximately pH 6 using 1 M sodium acetate and subjected to dialysis against 9 L of demineralized water five times using 3500 MWCO dialysis membranes (44310.02, MEMBRA-CEL, Serva). The solution was passed through 0.20 μm filters (Acrodisc 25 mm Supor STRL, Pall Corporation) before being stored at −20 °C.

#### 2.1.2 Solubilized elastin by basic hydrolysis (ELN-B)

3.6 g of purified elastin fibers were treated with 90 mL of 1 M KOH in an 80 % aqueous ethanol solution at 37 °C for 8 h under intermittent agitation until complete dissolution was achieved. The resulting solution was centrifuged at 15,000×*g* for 20 min at 4 °C to remove insoluble matter. The supernatant was neutralized using perchloric acid (HClO_4_), leading to the precipitation of potassium perchlorate (KClO_4_), which was subsequently eliminated by a second centrifugation step under identical conditions. The clarified supernatant underwent a dialysis process against 9 L of demineralized water five times using 3500 MWCO membranes. The final purified hydrolysate was filtered through 0.20 μm membranes and preserved at −20 °C.

#### 2.1.3 Characterization of solubilized elastins

Solubilized elastins were characterized as previously described [32]. The molecular weights were assessed using glycine-SDS-PAGE and size-exclusion chromatography. For SDS-PAGE analysis, solubilized elastin was heated in a reducing buffer at 100 °C for 5 min before being resolved on 10 % polyacrylamide gels. Protein bands were visualized using Coomassie Brilliant Blue staining SDS-PAGE. Size-exclusion chromatography was carried out using HiPrep 26/60 Sephacryl S-100 HR and S-400 HR columns (Cytiva) on an ÄKTA FPLC explorer system (Amersham Biosciences). Quantification of primary amine groups was performed via the 2,4,6-trinitrobenzene sulfonic acid (TNBS) assay as described by Buttafoco et al. [33] Absence of endotoxin contamination was confirmed using the ToxinSensor™ Chromogenic LAL endotoxin assay kit (Genscript) following the manufacturer's protocol.

### 2.2 Type I collagen scaffolds with solubilized elastins

#### 2.2.1 Preparation of the scaffolds

Porous non-crosslinked collagen scaffolds (COL) were fabricated by dispersing 0.8 % (w/v) bovine tendon-derived collagen fibrils into 0.25 M acetic acid and allowing them to swell overnight at 4 °C under continuous stirring. To incorporate solubilized elastins into the scaffolds (COL:ELN-A and COL:ELN-B), two collagen-to-elastin ratios – 97:3 and 95:5, resembling human skin – were tested. This involved dissolving either 0.024 % or 0.040 % (w/v) of ELN-A or ELN-B in 0.25 M acetic acid, followed by the addition of type I collagen fibrils at 0.776 % or 0.760 % in 0.25 M acetic acid, respectively. The resulting suspension was mixed thoroughly, swollen overnight at 4 °C, homogenized, poured into molds (4 mL per 960 mm^2^, 6-well plate), and freeze-dried using a Lyoph-pride 03 system (ilShin Biobase Europe).

Chemical crosslinking was carried out for 3 h using a solution containing 33 mM 1-ethyl-3-(3-dimethylaminopropyl)carbodiimide (EDC) and 6 mM N-hydroxysuccinimide (NHS) in a 50 mM 2-morpholinoethanesulfonic acid (MES) buffer with 40 % ethanol at pH 5.0. Then, scaffolds were washed sequentially with 0.1 M Na_2_HPO_4_, 1 M NaCl, and 2 M NaCl, followed by demineralized water rinses until the conductivity dropped below 200 μS (VWR, HCO 304). The final scaffolds were lyophilized and cut into ⌀12 mm discs for *in vitro* and *in vivo* studies.

#### 2.2.2 Characterization of the scaffolds

The scaffold morphology was examined via scanning electron microscopy (SEM). Dry samples were fixed onto metallic stubs with double-sided carbon tape, sputter-coated with gold for 60 s using a Polaron E5100 SEM coating system (Quorum Technologies), and imaged with a Zeiss Sigma 300 Field Emission Scanning Electron Microscope at 3 kV accelerating voltage.

For elastin visualization, scaffolds were embedded in Tissue-Tek O.C.T. compound (Sakura Finetek Europe), rapidly frozen on dry ice, and stored at −80 °C before sectioning. Thin sections (7 μm) were prepared using a Microm HM 550 VP cryostat (Thermo Scientific) at −20 °C and left to air dry overnight at room temperature. To block non-specific binding, samples were incubated with 0.5 % bovine serum albumin (BSA) in phosphate-buffered saline (pH 7.4) for 30 min. Elastin peptides were stained using a monoclonal elastin antibody (1:200, ab9519, Abcam) for 1 h, and incubated with biotinylated goat anti-mouse IgG (1:200, Vector Laboratories, BA-9200-1.5) for 1 h at room temperature in 0.5 % BSA-PBS. The VECTASTAIN Elite ABC Kit (Vector Laboratories) was applied according to the manufacturer's instructions. Elastin was detected in red using the ImmPACT AEC (3-amino-9-ethylcarbazole) HRP substrate kit (Vector Laboratories) and mounted with VectaMount AQ aqueous mounting medium (Vector Laboratories). A combination of Verhoeff's Elastic and Masson's Trichrome (VEMT) staining was performed to visualize collagen in blue and elastin in dark blue or black [34]. Imaging was conducted using a whole-slide scanner (3DHISTECH, Budapest, Hungary) with CaseViewer 2.4 software.

The crosslinking efficiency was assessed by quantifying the reduction in primary amine groups using the TNBS assay. For differential scanning calorimetry (DSC) analysis, approximately 1 mg of each sample was sealed in Tzero pans (TA Instruments) with hermetic lids and heated from 1 °C to 220 °C at a rate of 5 °C/min using a TA Q1000 DSC system (TA Instruments) equipped with an RCS40 cooler and Universal Analysis software. The onset temperature (T_onset_) was recorded to determine the denaturation temperature (T_d_) of collagen.

### 2.3 *In vitro* experiments with primary human fibroblasts

#### 2.3.1 Experimental design

Human dermal fibroblasts were obtained from three distinct origins: healthy adult skin, burn wound tissue (eschar), and fetal skin, following established protocols [35,36]. Cells were isolated from three different donors per tissue type, nine donors in total. Adult fibroblasts were obtained from skin samples of patients undergoing elective surgery at the Department of Plastic and Reconstructive Surgery, Red Cross Hospital (Beverwijk, NL). Eschar samples were collected from burn patients undergoing debridement between post-burn days 13 and 19 at the Red Cross Hospital. Fetal fibroblasts were derived from fetal skin obtained following pregnancy termination between gestational weeks 16 and 22, with written informed consent from the donors at the Center for Contraception, Abortion, and Sexuality (CASA, Leiden and The Hague, NL). Use of anonymized residual tissue was approved via the informed opt-out protocol, following national ethical guidelines (https://www.coreon.org/, accessed on November 23, 2020) and institutional privacy regulations.

Fibroblasts were maintained in fibroblast culture medium consisting of DMEM supplemented with 10 % fetal bovine serum (FBS), 5 U/mL penicillin and 5 μg/mL streptomycin, and 1 % GlutaMAX. Cultures were incubated at 37 °C in a 5 % CO_2_ atmosphere. For scaffold seeding, 100,000 fibroblasts (passages 3–4) were seeded on pre-wetted scaffold in triplicate per donor, using 24-well suspension culture plates (Cellstar). Media changes were performed on days 3, 7, and 11. On day 14, samples were collected and preserved for analysis by one of three methods: (1) fixed in Kryofix solution (50 % ethanol, 3 % PEG300) for histology, (2) frozen at −80 °C in cryotubes for SDS-PAGE and Western blotting, or (3) stored in TRIzol™ reagent (Invitrogen) at −80 °C for RNA extraction.

#### 2.3.2 Evaluation of cellular response and phenotype

Samples in Kryofix were embedded in paraffin, sectioned at 5 μm, and stained with hematoxylin and eosin (H&E). Processed sections were imaged using CaseViewer 2.4 software.

Protein expression was assessed via Western blotting, separated on 10 % SDS-PAGE gels. The primary antibody against α-smooth muscle actin (α-SMA) was applied at a 1:2000 dilution (clone 1A4, A-2547), alongside anti-GAPDH at a 1:1000 dilution (clone 14C10, ID218, Cell Signaling Technology). Secondary antibodies included goat polyclonal anti-mouse IgG (IRDye 800CW, 1:10,000, LI-COR Biotechnology, Lincoln) and goat anti-rabbit polyclonal IgG (IRDye 680CW, 1:15,000, LI-COR Biotechnology). Blots were analyzed using the Odyssey CLx imaging system (LI-COR).

RNA was extracted from TRIzol-preserved samples. Briefly, scaffolds were disrupted using a pipette tip, incubated at room temperature for 20 min, and centrifuged at 12,000×*g* at 4 °C. The supernatant was collected, and RNA was purified using the RNeasy Mini Kit (74106, Qiagen, Hilden, DE) with an RNase-free DNase set (79254, Qiagen). RNA yield and purity were assessed using a NanoDrop spectrophotometer (Thermo Scientific). Gene expression was quantified via reverse transcription-quantitative PCR (RT-qPCR), following the method described by Oostendorp et al. [37]. Target genes included ACTA2, ELN, COL1A1, with GAPDH and YWHAZ as reference genes (Table S1). Relative gene expression to COL scaffolds with adult fibroblasts (ΔΔCq) was calculated using CFX Maestro™ software (Bio-Rad), normalizing target gene expression to the reference genes.

### 2.4 *In vivo* evaluation of scaffolds

#### 2.4.1 Animal ethics and study approval

All surgical procedures were performed at the Radboudumc Animal Research Facility (Nijmegen, NL) after the Dutch Central Committee on Animal Research and the local Ethical Committee on Animal Research of the Radboud University approved the study under project license AVD10300202317189 and protocol 2023-0016-003, respectively.

#### 2.4.2 Animal model setup and scaffold implantation protocol

Five male Wistar subtype Crl:WI(WU) rats (3 months old, 250–400 g) were housed in groups of two or three per cage to avoid solitary housing. Upon arrival, the rats were labelled using ear punches and acclimated for 1–2 weeks before surgery through handling and training (Fig. S1). Rats were provided standard pellet food and water *ad libitum*.

On the day of surgery, anesthesia was used with 1.5–2.5 % isoflurane, analgesia was administered via carprofen subcutaneous injections (0.1 mL/100 g), and eye cream (Opthosan) was applied to prevent dryness. The dorsal area was prepared by trimming the fur with clippers and removing residual hair with depilatory cream (Veet), followed by disinfection with a povidone-iodine solution (Betadine, Kuinre, The Netherlands). Four full-thickness wounds (12 mm diameter) were created on the back of each rat using a biopsy punch (220701, SMI AG) and curved scissors. Untreated wounds (without any biomaterial) and wounds treated with COL served as internal controls, while COL:ELN-A (95:5) and COL:ELN-B (95:5) scaffolds were applied to the remaining two wound sites. Wounds on the back were labelled A-D for blinding, and the four treatments were allocated in a randomized order. Sterile wetted scaffolds were placed into the wounds and sutured using eight resorbable sutures (100L6P, Monocryl™ 4-0, Ethicon). The wounds were then covered with paraffin gauze (Jelonet, 7403, Smith & Nephew PLC), silicone dressing (Mepilex Border Flex Lite, 581277, Mölnlycke), elastic bandages (10009403914, PetFlex or elastic fixation bandage from Kruidvat), and adhesive plaster (250, Leukoplast®). Postoperative analgesia was administered via carprofen injections (0.1 mL/100 g) for three consecutive days. Wound dressings were replaced 1–3 times per week (when needed). Rats received booster food (V1185; ssniff Spezialdiäten) and sunflower seeds for 2–3 weeks after the operation.

Digital photographs were taken at multiple timepoints over a 7-week period to monitor wound healing. On day 56, the rats were euthanized using CO_2_ inhalation, and final digital photos of the wounds were taken. Wound sites were excised and bisected. One half was fixed in 4 % paraformaldehyde (PFA) in phosphate buffer pH 7.4 (PB) overnight at 4 °C and transferred to 1 % PFA in PB for storage prior to further analysis. The other half was embedded in TissueTek, frozen in liquid nitrogen and stored at −80 °C.

#### 2.4.3 Macroscopic and microscopic evaluation of wound healing

The final wound contraction was evaluated through the analysis of digital images captured immediately post-surgery (day 0) and at the last assessment point (day 56). Quantification of the wound area was performed using ImageJ software, with calibration of the measurement scale based on the ruler included within each image. The formula used to calculate the wound contraction was as follows:

$$Wound\;contraction\;\left(\%\right)=\frac{S_{0}-S_{56}}{S_{0}}\cdot 100\%$$

where

S_0_ = Initial wound area at day 0; S_56_ = Wound area at day 56.

For histological analysis, PFA-fixed samples were embedded in paraffin, processed, and cut into 5 μm sections. Sections were stained with H&E, followed by digital scanning with a whole-slide scanner and accompanying CaseViewer 2.4 software. H&E staining was used to measure the epidermal thickness in both wounded and unwounded skin, as an average for five regions per rat. The wound area was identified by the presence of short, loosely organized collagen fibers.

Elastin was labelled with rabbit anti-elastin antibody (AB2039, Chemicon, 1:100), followed by incubation with a biotinylated donkey anti-rabbit IgG (Jackson ImmunoResearch, 711-067-003, 1:200). The VECTASTAIN Elite ABC kit and ImmPACT AEC HRP substrate kit were used according to the manufacturer's instructions. Visualization of α-SMA was performed similarly, using a mouse monoclonal anti-α-SMA antibody (A2547, 1:200) and a biotinylated goat anti-mouse IgG (Vector Laboratories, BA-9200, 1:200). The stained sections were semi-quantitatively evaluated by two independent researchers (RK, NA) for signs of elastin deposition, angiogenesis, fibrotic healing, and foreign body reaction to the remaining scaffold material. Scores ranged from 0 (absent) to 2 (clearly present) with substantial agreement coefficients (weighed Cohen's Kappa ≥0.75).

An upright TrimScope II multiphoton microscope (LaVision BioTec, a Miltenyi Biotec company, Bielefeld, Germany) equipped with a 16 x 0.8 NA water immersion objective (CFI75 LWD 16X W, Nikon, Japan) was used to generate Second Harmonic Generation (SHG) and two-photon autofluorescence images from fixed rat-skin 5 μm tissue slices. A tunable Ti:Sa laser (Chameleon Ultra II, Coherent, CA, USA) tuned to 840 nm was used to excite the samples, with an average power of 14 mW under the objective. The microscope was equipped with a 6-channel non-descanned epi-detection port close to the objective. Emission was split with a 560lp followed by a 506lp dichroic mirror, and subsequently bandpass filtered with 417/60 (SHG) and 525/50 (autofluorescence). Emissions were detected with GaAs PMT detectors (H7422A-40, Hamamatsu, Hamamatsu city, Japan). Samples were imaged in bi-directional scanning mode, with a 624 μm scanfield, a 0.58 μm pixel size, and a pixel dwell time of 1.05 μs. Z-stacks were performed with a 3 μm step size. H&E stained sections combined with SHG images were used to semi-quantitatively assess newly formed collagen.

### 2.5 Statistical analysis

Statistical analyses were conducted using GraphPad Prism version 10.4.1. Relative measurements, normalized to the control condition, were evaluated using a one-sample *t*-test. Group comparisons were performed with Brown-Forsythe and Welch ANOVA, followed by Dunnett's T3 multiple comparisons test. Differences in gene expression levels between scaffold types and fibroblast types were tested using two-way ANOVA, followed by Tukey's multiple comparisons test. Statistical significance was defined as a p-value <0.05, corresponding to a 95 % confidence interval. To assess the level of agreement between the evaluators for semi-quantitative analysis, a weighted Cohen's Kappa was calculated using R version 4.2.2, with values ≥ 0.75 indicating substantial agreement. The weighting accounted for the severity of disagreement, meaning that a score difference between 0 and 2 reflects a greater discrepancy than between 1 and 2.

## 3 Results

### 3.1 Characterization of collagen-elastin scaffolds

As previously reported [32], different chemical solubilization methods for insoluble elastin resulted in distinct molecular weights and primary amine groups. Briefly, the peak molecular mass of ELN-A was approximately 39 kDa, and ELN-B was around 30 kDa, while quantification of primary amine groups resulted in values of 395 ± 77 nmol per mg for ELN-A and 214 ± 28 nmol per mg for ELN-B.

**Fig. 1:**
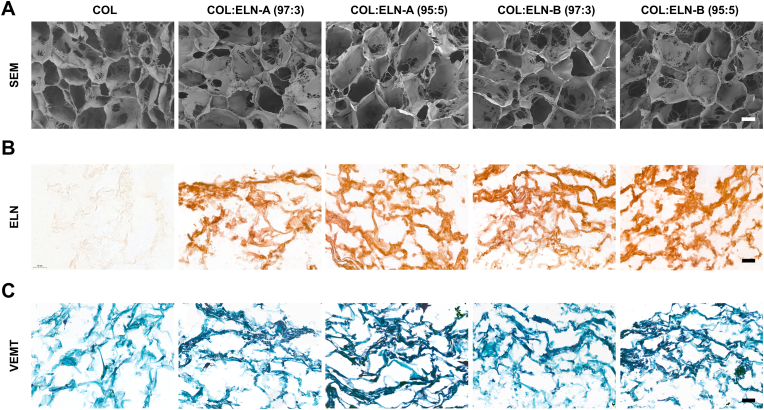
Microscopic imaging of collagen and collagen-elastin scaffolds. A) Scanning electron microscope (SEM) images of cross-sections of the scaffolds. B) Elastin(ELN)-stained sections of the scaffolds, highlighting elastin in brown. C) Verhoeff Elastic Masson Trichrome (VEMT) staining of the scaffolds, highlighting collagen in blue and elastin in dark-blue/black. Scale bars are 50 μm. COL = type I collagen scaffold, COL:ELN-A = type I collagen scaffold supplemented with ELN-A, COL:ELN-B = type I collagen scaffold supplemented with ELN-B. (For interpretation of the references to colour in this figure legend, the reader is referred to the Web version of this article.)

The collagen-elastin scaffolds were fabricated as white, soft, and porous biomaterials. Scanning electron microscopy confirmed that the covalent binding of soluble elastins with EDC/NHS did not alter the structural organization of the collagen matrix, as all scaffolds exhibited similar pore morphology (Fig. 1A). Immunohistochemical staining visualized elastin in brown, and VEMT staining highlighted collagen in blue and elastin in dark-blue/black. Both stainings demonstrated a successful incorporation of solubilized elastins and their uniform distribution throughout the scaffold (Fig. 1B–C).

Table 1 summarizes the biochemical characterization of scaffolds before and after chemical crosslinking. In the non-crosslinked state, collagen-only scaffolds contained the highest concentration of primary amine groups. Following crosslinking, all scaffold types showed amine content of 123 ± 4 nmol per mg of scaffold, resulting in comparable crosslinking efficiencies of 49 ± 2 %. Chemical crosslinking facilitated the covalent attachment of soluble elastin to insoluble type I collagen as well as enhanced scaffold stability. This was reflected in a significant increase in denaturation temperature (Td), with collagen and collagen-elastin scaffolds exhibiting respective increases of 19.3 ± 1.2 °C and 20.9 ± 0.5 °C. Furthermore, denaturation enthalpy analysis indicated that COL:ELN-B scaffolds maintained the highest protein structural integrity, regardless of crosslinking. In contrast, COL:ELN-A scaffolds showed reduced enthalpy values after crosslinking, possibly indicating differences in their secondary and tertiary structures compared to COL:ELN-B scaffolds.

**Table 1:** Characteristics of collagen and collagen-elastin scaffolds.

Scaffold	Amine group content (nmol/mg scaffold)		Denaturation temperature Td (°C)		Denaturation enthalpy ΔH_D_ (J/g)	
Crosslinked with EDC/NHS	–	+	–	+	–	+
COL	267 ± 51	126 ± 6∗	54.7 ± 1.2	74.0 ± 0.2∗	1.5 ± 0.3	1.9 ± 0.3
COL:ELN-A (97:3)	238 ± 34	122 ± 12∗	52.0 ± 0.4	73.2 ± 0.5∗	2.0 ± 0.1	1.6 ± 0.2
COL:ELN-A (95:5)	247 ± 38	126 ± 7∗	52.5 ± 0.8	72.5 ± 0.0∗	2.4 ± 0.2	1.9 ± 0.2
COL:ELN-B (97:3)	245 ± 30	119 ± 9∗	52.7 ± 0.3	73.9 ± 0.1∗	2.0 ± 0.2	2.3 ± 0.1
COL:ELN-B (95:5)	263 ± 33	124 ± 9∗	52.7 ± 0.8	72.9 ± 0.1∗	2.2 ± 0.1	2.5 ± 0.1

n = 3 ± SD. ∗ Compared to non-crosslinked counterpart, p < 0.001.

### 3.2 *In vitro* assessment of collagen-elastin scaffolds

To determine whether biomaterial composition influences the regenerative or fibrotic response, fibroblasts from different sources were used. We selected skin fibroblasts derived from adult, eschar, and fetal tissues.

**Fig. 2:**
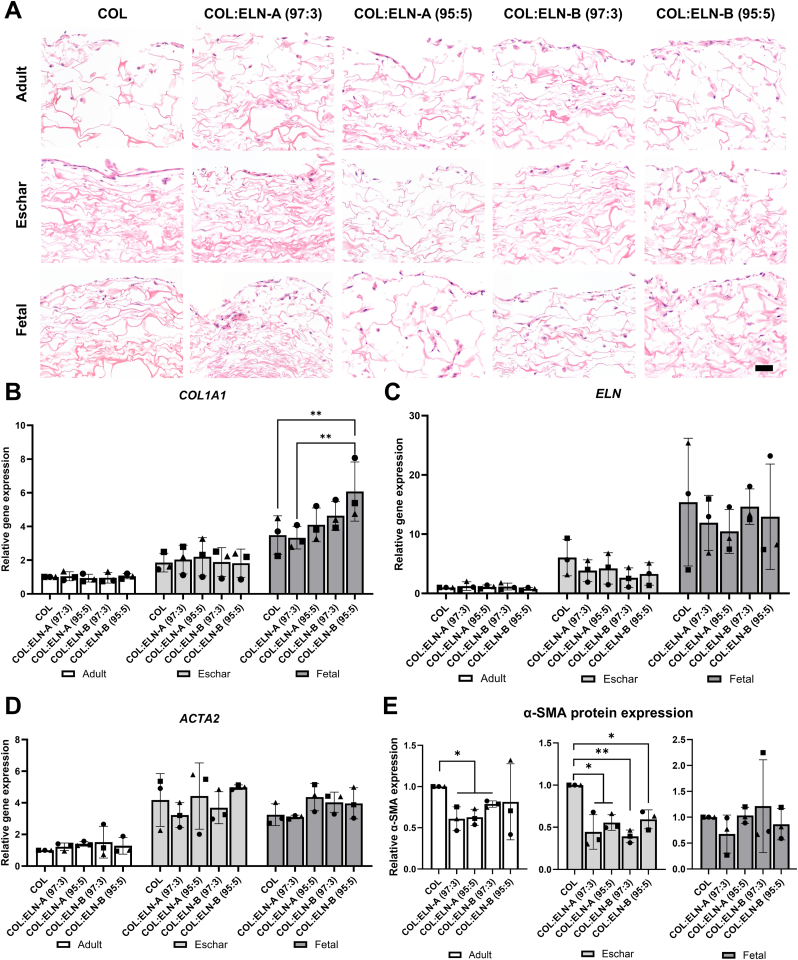
Results from cell culture experiments of primary human dermal fibroblasts (adult, eschar, and fetal) in the presence of scaffolds (day 14, n = 3). A) Cellular localization within scaffolds analyzed through H&E staining. Scale bar is 50 μm. B-D) Comparative gene expression levels of *COL1A1*, *ELN*, and *ACTA2* across three fibroblast types, normalized to the mean expression of adult fibroblasts cultured on COL scaffolds. Differences in gene expression levels between scaffold types and fibroblast types were tested using two-way ANOVA, followed by Tukey's multiple comparisons test (α = 0.05). Only statistically significant differences between scaffolds within the same fibroblast type are shown, while full pairwise comparisons are provided in Fig. S3 E) Relative expression of α-SMA based on intensities of the bands on Western blot, normalized to COL scaffolds for the same type of fibroblasts. Individual donors are represented by different symbols as ● donor 1/4/7, ■ donor 2/5/8, and ▲ donor 3/6/9. Error bars represent standard deviation; ∗p < 0.05, ∗∗p < 0.01, ∗∗∗p < 0.001. COL = type I collagen scaffold, COL:ELN-A = type I collagen scaffold supplemented with ELN-A, COL:ELN-B = type I collagen scaffold supplemented with ELN-B, COL1A1 = alpha-1 type I collagen, ELN = elastin, ACTA2 = smooth muscle actin alpha 2, α-SMA = α-smooth muscle actin.

H&E staining showed that more fetal fibroblasts were present on the scaffolds after two weeks of culture (Fig. 2A), followed by adult and eschar cells, which correlated with the amount of isolated RNA (Table S2). Fetal and adult cells showed slightly higher migration in both scaffold types than eschar cells, which mostly remained on the surface (Fig. 2A). In terms of contractile capacity, the scaffold size remained consistent at 1.1–1.2 cm^2^ (Fig. S2, Table S3).

After 14 days of culture, the expression of type I collagen (*COL1A1*) and elastin (*ELN*) genes was assessed by RT-qPCR analysis (Fig. 2B–C and Fig. S3A–B). Across three fibroblast types, the group of adult cells exhibited the lowest expression levels, whereas the group of eschar fibroblasts demonstrated significantly higher expression, likely reflecting an active ECM production state (Fig. S4). The group of fetal fibroblasts exhibited the highest gene expression for *COL1A1* and *ELN*. In fetal fibroblasts, COL:ELN-B (95:5) scaffolds resulted in higher *COL1A1* gene expression compared to COL and COL:ELN-A (97:3) (Fig. 2B). Significantly elevated gene expression was observed on the same scaffolds with adult compared to fetal fibroblasts (Fig. S3A–B).

To evaluate myofibroblast presence, α-SMA expression was analyzed at gene (*ACTA2*; Fig. 2D) and protein level (Fig. 2E and Fig. S4D, S5). For all five scaffolds, adult fibroblasts demonstrated significantly lower *ACTA2* expression compared to eschar and fetal cells (Fig. S3C). α-SMA expression assessed through Western blot showed the same trend, with higher expression in fetal (p < 0.001) and eschar (p = 0.064) fibroblasts compared to adult cells (Fig. S4D). For adult and eschar cells, α-SMA protein expression was reduced in COL:ELN-A (97:3 and 95:5) and COL:ELN-B (97:3), compared to collagen only. Additionally, COL:ELN-B (95:5) decreased α-SMA expression in eschar cells.

### 3.3 Analysis of wound healing with collagen-elastin scaffolds *in vivo*

**Fig. 3:**
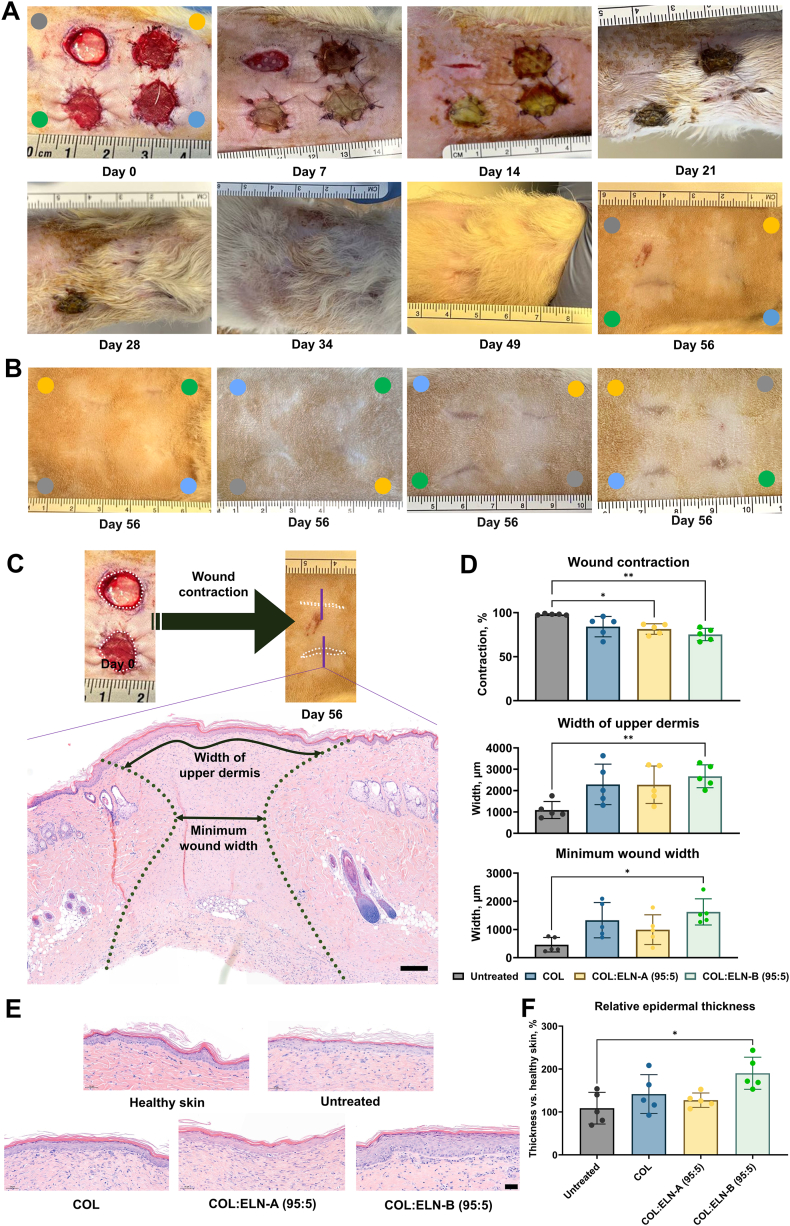
*In vivo* assessment of the scaffolds in a rat model (n = 5). A) Representative digital images of the healing process of the wounds throughout the experiment. ● – untreated wound, ● – type I collagen scaffold (COL), ● – type I collagen scaffold supplemented with ELN-A (COL:ELN-A (95:5)), ● – type I collagen scaffold supplemented with ELN-B (COL:ELN-B (95:5)). B) Healed skin regions in the other four rats at day 56. Scale bars in centimetres. C) Representative H&E stained skin sections on day 56 with wound area highlighted with grey dotted lines, and arrows showing how the newly formed upper dermis and minimum wound width were measured. Scale bar is 200 μm. D) Calculated macroscopic wound contraction (top), width of the newly formed upper dermis (middle), and minimum wound width (bottom). E) Representative H&E stained sections showing epidermal thickness of healthy skin and four experimental groups. F) Epidermal thickness within four treatment groups, expressed relative to adjacent healthy skin (%). Scale bar is 50 μm. Error bars represent standard deviation; ∗p < 0.05, ∗∗p < 0.01.

Three types of biomaterials were tested in excisional wounds in a rat model: COL, COL:ELN-A (95:5) and COL:ELN-B (95:5), with an untreated wound as a control. The four wounds exhibited distinct healing trajectories following a similar morphological progression. The wound consistently decreased in size over time, transitioning from a circular shape to an oval form, and ultimately resolving into a linear scar (Fig. 3A–B). Among the groups, untreated wounds displayed the fastest closure, reaching near-complete closure by day 14, with approximately 99 % wound contraction at the end of the observation period.

The extent of macroscopic wound contraction followed the trend: untreated wound > COL > COL:ELN-A > COL:ELN-B (Fig. 3A–D). Although the differences in contraction between scaffolds were not statistically significant, a significant reduction in wound contraction was observed when comparing untreated wounds to those treated with scaffolds supplemented with elastin hydrolysates (Fig. 3D). Results from *in vitro* experiments demonstrating reduced α-SMA expression in healthy and wounded (eschar) adult skin fibroblasts cultured on elastin-containing scaffolds suggest that the decreased wound contraction observed with collagen-elastin scaffolds may be attributed, at least in part, to suppressed myofibroblast activation. Among the scaffolds, COL:ELN-B (95:5) demonstrated the most pronounced reduction in visual contraction *in vivo*.

The wounded area can be seen with H&E staining, characterized by thinner and smaller collagen fibrils (Fig. 3C and Fig. S7). All the wounds exhibited a concave wound geometry under histological examination (grey dotted lines in Fig. 3C). Notably, skin sections were obtained from the central perpendicular plane of the wounds, despite the middle region not always representing the widest part of the wound. Wound contraction was additionally quantitatively assessed by measuring the width of the newly formed upper dermis and the minimum wound width (Fig. 3C–D). All biomaterials demonstrated higher width values of newly formed ECM regions, while COL:ELN-B (95:5) biomaterials resulted in the largest width values and were significantly different compared to the untreated wounds (Fig. 3D).

Throughout the study, wounds treated with COL scaffolds generally closed more rapidly, while those treated with COL:ELN-B (95:5) scaffolds exhibited the slowest healing, with closure times extending up to 35 days. Additionally, wounds located in the upper body regions of the rats were less affected by movement-related distortion compared to those in the lower regions, highlighting the impact of anatomical location on healing outcomes. To minimize positional bias, treatment conditions were randomly assigned to different wound locations across animals, ensuring that no rat received the same allocation pattern.

H&E staining also showed other aspects of wound healing, including re-epithelialization, cellular infiltration, and the amount of scaffold remnants. Measurement of epidermal thickness revealed that the COL:ELN-B (95:5) scaffold showed a significant increase in relative epidermal thickness compared to adjacent healthy skin (Fig. 3E–F and Fig. S6). Untreated wounds, COL and COL:ELN-A scaffolds resulted in comparable epidermal thickness values.

**Fig. 4:**
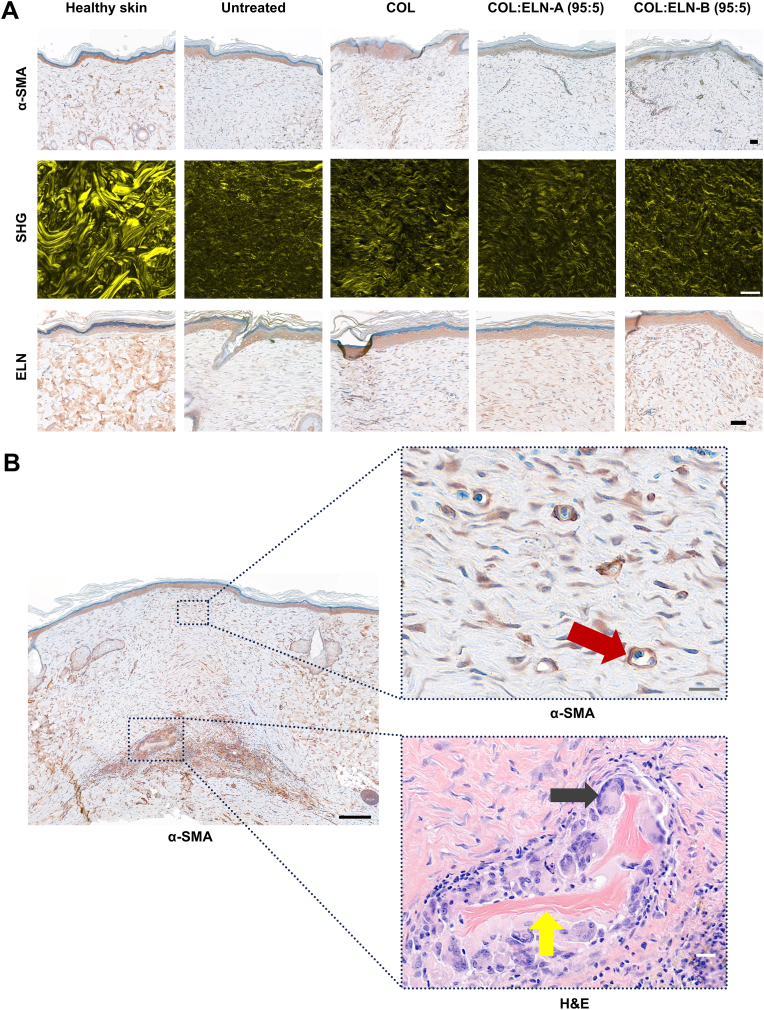
Microscopic examination of healthy and healed rat skin at day 56. A) Representative sections stained for α-SMA, in brown. Collagen fibers visualized with SHG using multiphoton microscopy. Sections stained for elastin, in brown. Scale bars are 50 μm. B) Section stained for α-SMA, presenting an area with strong positive staining, co-localizing with residual scaffold fragments (yellow arrow), surrounded by foreign body giant cells (grey arrow) (magnified area, H&E). α-SMA staining also highlights neovascularization (magnified area, red arrow). Black scale bar is 200 μm, grey and white scale bars are 20 μm. COL = type I collagen scaffold, COL:ELN-A = type I collagen scaffold supplemented with ELN-A, COL:ELN-B = type I collagen scaffold supplemented with ELN-B, α-SMA = α-smooth muscle actin, SHG = second harmonic generation, ELN = elastin, H&E = hematoxylin and eosin. (For interpretation of the references to colour in this figure legend, the reader is referred to the Web version of this article.)

All experimental groups showed increased cellular infiltration in the wounded areas compared to healthy skin, likely due to a transient inflammatory response and ongoing tissue remodeling. α-SMA staining highlighted the formation of new blood vessels and showed weaker overall intensity compared to healthy skin (Fig. 4A–B). α-SMA-positive staining around capillaries was interpreted as a marker of vascular maturation, reflecting the presence of pericytes surrounding endothelial cells, which contributed to vessel stabilization and functional integrity. Neovascularization was observed across all treatment groups within the wounded regions (Table 2). When scaffold remnants persisted within the dermis, they were distinctly visible through both α-SMA and H&E staining, and induced a foreign body reaction (Fig. 4B). Notably, a strong localized α-SMA positive signal in fibroblasts, coupled with high cellular infiltration and scaffold fragments surrounded by giant cells, suggested active scaffold degradation and local remodeling around the degrading material. The most frequent and apparent presence of COL:ELN-B (95:5) remnants coincided with the longest healing time for this treatment group.

**Table 2:** Comparable analysis of rat skin tissue sections at day 56 after wounding.

	Untreated	COL	COL:ELN-A (95:5)	COL:ELN-B (95:5)
**Scaffold remnants**	N/A	0.6 ± 0.8	0.7 ± 0.7	1.1 ± 0.5
**Neovascularization**	0.6 ± 0.5	1.4 ± 0.8	1.2 ± 0.7	1.5 ± 0.4
**Collagen**	0.5 ± 0.2	1.2 ± 0.6	1.0 ± 0.4	1.3 ± 0.2∗∗
**Elastin**	0.2 ± 0.4	1.1 ± 0.2∗	1.4 ± 0.4∗	1.4 ± 0.5∗

The presence of a localized area of strong α-SMA positive staining in response to the presence of residual scaffold fragments and neovascularization was scored from 0 (absence) to 2 (clear detection). Similarly, collagen and elastin deposition were assessed through H&E/SHG and elastin staining, respectively, and scored on the same 0 (absence) to 2 (most detection) scale. n = 5, mean ± SD. N/A = not applicable.

∗ compared to untreated wound, p < 0.05; ∗∗p < 0.01.

Collagen fibers were also visualized using second harmonic generation (SHG) (Fig. 4A). In wounded areas, SHG detected weaker collagen signals and revealed finer thickness of newly formed collagen fibrils (Fig. S7). Semi-quantitative assessment revealed a significant increase in collagen deposition in wounds treated with the COL:ELN-B scaffold compared to untreated wounds. Elastin immunostaining showed the strongest positive signal in healthy skin, while all scaffold-treated groups (including collagen-only scaffolds) exhibited weaker but consistent signals, likely due to the formation of new elastic fibers. Among the scaffold-treated groups, the COL:ELN-B (95:5) scaffold was associated with the least wound contraction and appeared to support more extensive deposition of collagen and elastic fibers, although deposition patterns were generally similar across all scaffolds and these differences were not statistically significant. In contrast, untreated wounds revealed the minimum collagen deposition and almost no presence of elastin. Two-photon excited autofluorescence imaging did not visualize elastic fibers but highlighted integrated scaffolds into the rat's dermis (Fig. S8).

## 4 Discussion

Elastin and elastin-based compounds have been extensively studied for their beneficial biological effects in wound healing, appearing in various forms such as insoluble elastic fibers [38], soluble tropoelastin (protein [39] or mRNA [40]), solubilized elastin peptides [22], and synthetic elastin peptides [41]. However, when considering their application in dermal biomaterial development, the primary candidates are elastic fibers (insoluble or solubilized) and recombinant tropoelastin protein. Among these, insoluble elastin derived from animal sources is the least favorable due to challenges such as calcification, poor solubility, and biological inertness [22,38]. While recombinant tropoelastin offers a promising alternative, its application in dermal biomaterials is complicated by limited stability, high production costs, and significant challenges in large-scale manufacturing [39,42,43]. Given these aspects, we opted for solubilized elastins, which offer distinct advantages over the aforementioned compounds and their limitations.

Solubilized elastin peptides can be categorized based on their preparation method: chemical hydrolysis (using acids or bases) or enzymatic digestion. Different enzymatically digested elastins have demonstrated potential in promoting ECM synthesis and dermal regeneration [19,20]. Although these findings originate from single publications by individual research groups, they provide important insights and a strong foundation for continued investigation. Alternatively, chemically hydrolyzed elastins have been more extensively studied, with demonstrated effects in cell cultures, animal models, and biomaterial formulations [24]. Among acidic hydrolysis methods, oxalic acid is the most commonly used because of its ability to generate solubilized elastin that closely mimics its native, insoluble counterpart [44,45]. Such a preparation was able to enhance elastin expression and improve dermal healing when incorporated into biomaterials [22,46,47]. Alkaline hydrolysis of elastin is typically performed with potassium hydroxide in an ethanol medium, which provides hydroxide ions for nucleophilic cleavage and may lead to β-elimination and deamination reactions (as indicated by an almost twofold reduction in primary amine groups in ELN-B compared to ELN-A). Such solubilized elastins obtained via alkaline hydrolysis have not been widely used in biomaterial formulations but have been reported to enhance mesenchymal cell adhesion to elastic fibers, stimulate the biosynthesis of adhesive proteins, and strengthen cell-elastin interactions [48]. Additionally, they have found applications in cosmetics due to their UV-protective properties and ability to improve skin elasticity [49,50].

We selected solubilized elastins derived from oxalic acid and potassium hydroxide treatments for further investigation and direct comparison in collagen-based scaffolds. To achieve covalent binding of soluble elastin to insoluble collagen fibers, we used EDC/NHS crosslinking [22]. A previous study has reported similar concentrations of primary amine groups in soluble elastin and collagen (approximately 300 nmol/mg [22]), while our materials showed slight variations: type I collagen contained 270 nmol/mg, ELN-A – 400 nmol/mg, and ELN-B – 200 nmol/mg. After crosslinking, the remaining available primary amine groups were approximately 125 nmol/mg across all formulations, similar to earlier findings of around 180 nmol/mg [22] and resulting in ∼50 % crosslinking. These discrepancies may be attributed to differences in methods for preparation of soluble elastin (stepwise oxalic acid hydrolysis vs. continuous oxalic acid and alkaline hydrolysis) and scaffold compositions (97:3, 9:1, 1:1 vs. 97:3, 95:5). Although mechanical testing was not performed on our biomaterials, previous studies have shown that collagen-elastin scaffolds crosslinked with genipin or EDC/NHS showed increased elasticity with the addition of elastin [29,51]. Elastin has also been reported to delay scaffold degradation [29]. In our pilot experiments with soluble elastin, we observed that a higher elastin content accelerated degradation of crosslinked scaffolds in the presence of collagenase, but delayed degradation in non-crosslinked ones (data not shown). Unlike prior study utilizing a 1:1 collagen-elastin ratio for *in vivo* experiments [22], we investigated formulations with 95:5 collagen-elastin ratios, which more closely resemble the elastin content in human skin [9].

Studies on the *in vitro* evaluation of collagen-solubilized elastin scaffolds are limited [29,30,51]. We observed that cell distribution, with higher densities closer to the seeded side, aligned with previously reported data [30,52]. In this study, we explored the potential for scarless healing by analyzing key genes (*COL1A1*, *ACTA2*, *ELN*) and α-SMA protein expression, as well as examining the responses of three distinct skin fibroblast sources, adult, eschar, and fetal cells, in collagen-elastin scaffolds. While these fibroblast types share some similarities, they exhibit unique behaviors in culture, reflecting their respective roles in wound healing and tissue development. Adult fibroblasts cultured on COL:ELN-A scaffolds showed lower α-SMA protein levels, indicating a potentially reduced fibrotic response. Notably, fetal fibroblasts demonstrated features typical of myofibroblasts and generated an environment resembling fibrosis [53], yet contributed to healing without contracture or scar formation [3]. Fetal fibroblasts exhibited higher α-SMA expression compared to adult fibroblasts in cell culture experiments, consistent with previous findings from monolayer cultures [54]. Furthermore, the upregulation of α-SMA at both the gene and protein level for eschar fibroblasts aligned with recent literature [55]. Nevertheless, cellular responses under fibrotic conditions induced by TGF-β may follow different patterns. For example, studies on collagen gels have shown that fetal fibroblasts exhibit reduced contractile capacity, characterized by lower cell proliferation and decreased expression of α-SMA and integrins [54]. Due to the stiffness of our crosslinked scaffolds and their resistance to cell-mediated contraction, along with the absence of TGF-β stimulation, scaffold size remained stable throughout the *in vitro* experiments. Our findings highlight that different fibroblast sources exhibit distinct biological behaviors, suggesting that each cell type may offer specific advantages depending on the wound context (e.g., incisional vs. burn injuries) or therapeutic application (e.g., in pediatric vs. adult patients).

Collagen-elastin scaffolds represent a valuable alternative to many current wound healing strategies by combining biomimicry with practical application [24]. Unlike synthetic polymers, which often lack the intrinsic biological resemblance [56], these scaffolds simulate the structural and functional properties of the dermis by providing the tensile strength of collagen together with the elasticity provided by elastin. Compared to cell-based therapies, which have issues with viability, retention, risk of tumor formation, and precise delivery within the wound environment [57,58], collagen-elastin scaffolds serve as supportive, biocompatible matrices that support proliferation and differentiation of host cells. While advanced tissue engineering techniques such as complex skin grafts, 3D bioprinting, aim to closely replicate full skin architecture [[59], [60], [61]], they are often constrained by high costs, technical complexity, and scalability challenges. In contrast, collagen-elastin scaffolds provide a more accessible, cost-effective platform that supports robust tissue regeneration without the need for complex structural design or cellular manipulation, making them also highly attractive for clinical translation.

In animal models, research on biomaterials composed of type I collagen and solubilized elastin has been primarily focused on oxalic acid hydrolysates [22,23,26,47,62]. When these hydrolysates were incorporated into non-crosslinked collagen scaffolds and applied to full-thickness wounds in Yorkshire pigs, the biomaterials degraded more slowly compared to collagen-only scaffolds [47]. Additionally, more newly formed ECM was observed within the biomaterial's pores, although there was a lower number of infiltrating fibroblasts and myofibroblasts [23,47]. Most importantly, collagen-elastin matrices significantly reduced wound contraction in pigs and provided the best esthetic outcomes [47]. In Sprague Dawley rats, subcutaneous implantation of EDC/NHS crosslinked collagen-elastin scaffolds showed that oxalic acid hydrolysates of elastin promoted angiogenesis, as indicated by type IV collagen staining [22]. These scaffolds also supported the formation of elastic fibers, with notable deposits of elastin and fibrillin-1, as well as a more evident collagen network composed of type I and type III collagen [22]. Full-thickness wound healing in Wistar rats with a double-layered skin substitute containing solubilized elastin was also found to promote significant angiogenesis while reducing myofibroblast presence during the early stages of healing [26]. By the end of the 112-day experiment, the tested skin substitute facilitated the formation of new elastic fibers and structures resembling skin appendages [26]. Despite these promising effects, the exact impact of solubilized elastin was not determined because of the scaffold's complex composition (type I collagen, heparin, solubilized elastin, dermatan sulfate, fibroblast growth factors 2 and 7, and vascular endothelial growth factor). Among the tested conditions in our experiments, COL:ELN-B scaffolds exhibited a prolonged wound healing response but the least wound contraction by day 56 post-surgery, although the differences were not statistically significant from other tested scaffolds. Additionally, we observed increased collagen and elastin deposition in collagen-elastin scaffolds, along with enhanced angiogenesis. These findings not only support the hypothesis that collagen-elastin scaffolds have beneficial effects on wound healing but also highlight the promising potential of the incorporation of the potassium hydroxide hydrolysate. While other methods for producing materials for wound healing may require high costs and laborious techniques (e.g., 3D bioprinting, stereolithography, selective laser sintering, and fused deposition modeling), Multiphoton microscopy offers a non-invasive approach for examining ECM structures in unstained tissue sections [63]. In this study, collagen was visualized through SHG, while elastic fibers were expected to be detected via TPEAF [64,65]. This technique eliminates the need for tissue excision, embedding, fixation, or staining, and can be applied to live animals [66,67], potentially reducing the number required for experiments. Although dermal elastic fibers were not clearly observed under the chosen TPEAF settings, we did detect autofluorescent signals in the scaffold-treated groups, which are likely associated with parts of biomaterials integrated into the rat skin.

Research on solubilized elastins involves several important considerations. Firstly, it is crucial to determine whether the entire hydrolysate or a specific fraction is used, as their differing molecular characteristics can influence the observed effects. Secondly, studies assessing elastogenic effects should clearly differentiate between elastin upregulation at the gene level and actual protein production. The formation of a well-organized elastic fiber network should be demonstrated, rather than the presence of isolated elastin deposits [68]. Furthermore, fluorescent tagging of solubilized elastins combined with antibody detection of rat's elastic fibers may be used to distinguish whether soluble elastin was incorporated into new elastic fibers or merely triggered elastogenesis without incorporation [69]. Thirdly, when testing biomaterials in animal models, selecting the appropriate species is essential. Despite their higher costs and relative complexity, larger animals such as pigs are preferred due to their closer resemblance to human skin [70,71]. Exploring alternative, milder crosslinking agents (e.g., genipin, transglutaminase, epigallocatechin gallate, tannic acid [72,73]) may promote faster scaffold resorption within wounded skin regions, potentially enhancing tissue regeneration and reducing the risk of an unwanted foreign body response [25]. In this context, evaluating the mechanical stability and biodegradation of these scaffolds, particularly at later timepoints, is crucial for understanding how long they persist in the tissue and how they influence cellular behavior and remodeling dynamics. Additionally, any comparisons to previously published animal studies are complicated by substantial variations in biomaterial composition, preparation methods, and species used.

## 5 Conclusions

Two differently prepared solubilized elastins were successfully integrated into collagen-based biomaterials and compared side-by-side. Among the three types of skin fibroblasts tested, fetal cells exhibited the highest expression of extracellular matrix-related genes and cellular infiltration within the scaffolds, followed by eschar-derived cells and healthy adult dermal fibroblasts. Compared to collagen-only scaffolds, adult and eschar fibroblasts cultured on collagen-elastin scaffolds demonstrated lower expression of α-SMA, a fibrotic marker, suggesting that the presence of elastin may suppress myofibroblast activation for these types of fibroblasts. In a rat full-thickness skin model, α-SMA staining did not suggest fibrotic responses but did indicate neovascularization and an ongoing degradation and clearance of scaffold remnants by the host tissue. *In vivo*, collagen scaffolds supplemented with elastin hydrolysate obtained by basic hydrolysis showed promising features associated with scarless healing, as evidenced by the lowest wound contraction, the most extensive newly formed ECM, and the highest degree of neovascularization.

## CRediT authorship contribution statement

**Roman Krymchenko:** Writing – review & editing, Writing – original draft, Visualization, Validation, Project administration, Methodology, Investigation, Formal analysis, Data curation, Conceptualization. **Nancy Avila-Martinez:** Writing – review & editing, Validation, Methodology, Formal analysis. **Merel Gansevoort:** Writing – review & editing, Validation, Methodology. **Gert-Jan Bakker:** Writing – review & editing, Validation, Methodology. **Madalena L.N.P. Gomes:** Writing – review & editing, Validation, Methodology. **Marcel Vlig:** Writing – review & editing, Validation, Methodology. **Elly M.M. Versteeg:** Writing – review & editing, Methodology. **Bouke K.H.L. Boekema:** Writing – review & editing, Validation, Supervision, Resources, Project administration, Methodology, Funding acquisition. **Toin H. van Kuppevelt:** Writing – review & editing, Validation, Supervision, Resources, Project administration, Methodology, Funding acquisition. **Willeke F. Daamen:** Writing – review & editing, Validation, Supervision, Resources, Project administration, Methodology, Funding acquisition.

## Funding

The work has received funding from the European Union's Horizon 2020 research and innovation programme under the 10.13039/100010665Marie Skłodowska‐Curie Grant Agreement No. 955722 (RK, NAM, MLNPG).

## Declaration of competing interest

The authors declare that they have no known competing financial interests or personal relationships that could have appeared to influence the work reported in this paper.

## Data Availability

Research data associated with this publication will be made available at the Radboud data repository under a RUMC-RA-DUA-1.0 license at DOI 10.34973/qs9v-vj42 upon publication of the manuscript.

## References

[1] Strodtbeck F. (2001). Physiology of wound healing, newborn infant. Nurs. Rev..

[2] Alonso L., Fuchs E. (2003). Stem cells of the skin epithelium. Proc. Natl. Acad. Sci..

[3] Buchanan E.P., Longaker M.T., Lorenz H.P. (2009). Chapter 6 fetal skin wound healing. Adv. Clin. Chem., Elsevier.

[4] Rivera A.E., Spencer J.M. (2007). Clinical aspects of full-thickness wound healing. Clin. Dermatol..

[5] Svensjö T., Pomahac B., Yao F., Slama J., Eriksson E. (2000). Accelerated healing of full-thickness skin wounds in a wet environment. Plast. Reconstr. Surg..

[6] Rittié L. (2016). Cellular mechanisms of skin repair in humans and other mammals. J. Cell Commun. Signal..

[7] Boyce S.T., Simpson P.S., Rieman M.T., Warner P.M., Yakuboff K.P., Bailey J.K., Nelson J.K., Fowler L.A., Kagan R.J. (2017). Randomized, paired-site comparison of autologous engineered skin substitutes and split-thickness skin graft for closure of extensive, full-thickness burns. J. Burn Care Res..

[8] Tavakoli S., Klar A.S. (2021). Bioengineered skin substitutes: advances and future trends. Appl. Sci..

[9] Sheikholeslam M., Wright M.E.E., Jeschke M.G., Amini-Nik S. (2018). Biomaterials for skin substitutes. Adv. Healthcare Mater..

[10] Rezvani Ghomi E., Nourbakhsh N., Akbari Kenari M., Zare M., Ramakrishna S. (2021). Collagen-based biomaterials for biomedical applications. J. Biomed. Mater. Res. B Appl. Biomater..

[11] Mathew-Steiner S.S., Roy S., Sen C.K. (2021). Collagen in wound healing. Bioengineering.

[12] Ferrario C., Rusconi F., Pulaj A., Macchi R., Landini P., Paroni M., Colombo G., Martinello T., Melotti L., Gomiero C., Candia Carnevali M.D., Bonasoro F., Patruno M., Sugni M. (2020). From food waste to innovative biomaterial: sea urchin-derived collagen for applications in skin regenerative medicine. Mar. Drugs.

[13] Schmelzer C.E.H., Duca L. (2022). Elastic fibers: formation, function, and fate during aging and disease. FEBS J..

[14] Cohen B.E., Geronemus R.G., McDaniel D.H., Brauer J.A. (2017). The role of elastic fibers in scar formation and treatment. Dermatol. Surg..

[15] Senior R.M., Griffin G.L., Mecham R.P., Wrenn D.S., Prasad K.U., Urry D.W. (1984). Val-Gly-Val-Ala-Pro-Gly, a repeating peptide in elastin, is chemotactic for fibroblasts and monocytes. J. Cell Biol..

[16] Morelli M.A.C., Bisaccia F., Spisani S., Biasi M.D., Traniello S., Tamburro A.M. (1997). Structure-activity relationships for some elastin-derived peptide chemoattractants. J. Pept. Res..

[17] Kamoun A., Landeau J.M., Godeau G., Wallach J., Duchesnay A., Pellat B., Hornebeck W. (1995). Growth stimulation of human skin fibroblasts by elastin-derived peptides, cell adhes. Commun. Now..

[18] Tajima S., Wachi H., Uemura Y., Okamoto K. (1997). Modulation by elastin peptide VGVAPG of cell proliferation and elastin expression in human skin fibroblasts. Arch. Dermatol. Res..

[19] Hinek A., Wang Y., Liu K., Mitts T.F., Jimenez F. (2005). Proteolytic digest derived from bovine Ligamentum Nuchae stimulates deposition of new elastin-enriched matrix in cultures and transplants of human dermal fibroblasts. J. Dermatol. Sci..

[20] Shiratsuchi E., Nakaba M., Yamada M. (2016). Elastin hydrolysate derived from fish enhances proliferation of human skin fibroblasts and elastin synthesis in human skin fibroblasts and improves the skin conditions. J. Sci. Food Agric..

[21] Groult V., Hornebeck W., Ferrari P., Tixier J.M., Robert L., Jacob M.P. (1991). Mechanisms of interaction between human skin fibroblasts and elastin: differences between elastin fibres and derived peptides. Cell Biochem. Funct..

[22] Daamen W.F., Nillesen S.T., Wismans R.G., Reinhardt D.P., Hafmans T., Veerkamp J.H., van Kuppevelt T.H. (2008). A biomaterial composed of collagen and solubilized elastin enhances angiogenesis and elastic fiber formation without calcification. Tissue Eng..

[23] Lamme E.N., de Vries H.J., van Veen H., Gabbiani G., Westerhof W., Middelkoop E. (1996). Extracellular matrix characterization during healing of full-thickness wounds treated with a collagen/elastin dermal substitute shows improved skin regeneration in pigs. J. Histochem. Cytochem..

[24] Daamen W.F., Veerkamp J.H., van Hest J.C., van Kuppevelt T.H. (2007). Elastin as a biomaterial for tissue engineering. Biomaterials.

[25] Boekema B., Vlig M., Damink L., Middelkoop E., Eummelen L., Bühren A., Ulrich M. (2014). Effect of pore size and cross-linking of a novel collagen-elastin dermal substitute on wound healing. J. Mater. Sci. Mater. Med..

[26] Nillesen S.T.M., Lammers G., Wismans R.G., Ulrich M.M., Middelkoop E., Spauwen P.H., Faraj K.A., Schalkwijk J., Daamen W.F., van Kuppevelt T.H. (2011). Design and in vivo evaluation of a molecularly defined acellular skin construct: reduction of early contraction and increase in early blood vessel formation. Acta Biomater..

[27] Jiménez Vázquez J., San Martín Martínez E. (2019). Collagen and elastin scaffold by electrospinning for skin tissue engineering applications. J. Mater. Res..

[28] Sawadkar P., Mandakhbayar N., Patel K.D., Buitrago J.O., Kim T.H., Rajasekar P., Lali F., Kyriakidis C., Rahmani B., Mohanakrishnan J., Dua R., Greco K., Lee J.-H., Kim H.-W., Knowles J., García – Gareta E. (2021). Three dimensional porous scaffolds derived from collagen, elastin and fibrin proteins orchestrate adipose tissue regeneration. J. Tissue Eng..

[29] Kamaruzaman N., Fauzi M.B., Tabata Y., Yusop S.M. (2023). Functionalised hybrid collagen-elastin for acellular cutaneous substitute applications. Polymers.

[30] Maitz J., Wang Y., Fathi A., Ximena Escobar F., Parungao R., van Zuijlen P., Maitz P., Li Z. (2020). The effects of cross-linking a collagen-elastin dermal template on scaffold bio-stability and degradation. J. Tissue Eng. Regen. Med..

[31] Daamen W.F., Hafmans T., Veerkamp J.H., van Kuppevelt T.H. (2005). Isolation of intact elastin fibers devoid of microfibrils. Tissue Eng..

[32] Krymchenko R., Pfirrmann M., van der Leeuw S., Avila-Martinez N., Versteeg E.M.M., Meuwese R.T.C., Vlig M., Verdoes M., Boekema B.K.H.L., van Kuppevelt T.H., Daamen W.F. (2025). Preparation, fractionation, and characterization of solubilized elastin and comparison of cellular response on fibroblasts and macrophages. Int. J. Biol. Macromol..

[33] Buttafoco L., Engbers-Buijtenhuijs P., Poot A.A., Dijkstra P.J., Daamen W.F., van Kuppevelt T.H., Vermes I., Feijen J. (2006). First steps towards tissue engineering of small-diameter blood vessels: preparation of flat scaffolds of collagen and elastin by means of freeze drying. J. Biomed. Mater. Res. B Appl. Biomater..

[34] O'Connor W.N., Valle S. (1982). A combination verhoeffs elastic and masson's Trichrome stain for routine histology. Stain Technol..

[35] van den Bogaerdt A.J., van Zuijlen P.P., van Galen M., Lamme E.N., Middelkoop E. (2002). The suitability of cells from different tissues for use in tissue-engineered skin substitutes. Arch. Dermatol. Res..

[36] Akershoek J.J., Vlig M., Talhout W., Boekema B.K.H.L., Richters C.D., Beelen R.H.J., Brouwer K.M., Middelkoop E., Ulrich M.M.W. (2016). Cell therapy for full-thickness wounds: are fetal dermal cells a potential source?. Cell Tissue Res..

[37] Oostendorp C., Geutjes P.J., Smit F., Tiemessen D.M., Polman S., Abbawi A., Brouwer K.M., Eggink A.J., Feitz W.F.J., Daamen W.F., van Kuppevelt T.H. (2021). Sustained postnatal skin regeneration upon prenatal application of functionalized collagen scaffolds. Tissue Eng..

[38] Daamen W.F., Nillesen S.T.M., Hafmans T., Veerkamp J.H., van Luyn M.J.A., van Kuppevelt T.H. (2005). Tissue response of defined collagen–elastin scaffolds in young and adult rats with special attention to calcification. Biomaterials.

[39] Mitzmacher M.G., Mithieux S.M., Weiss A.S., Hee C.K., Daniels R. (2020). Novel recombinant tropoelastin implants restore skin extracellular matrix. J. Drugs Dermatol. JDD : J. Drugs Dermatol. JDD.

[40] Lescan M., Perl R.M., Golombek S., Pilz M., Hann L., Yasmin M., Behring A., Keller T., Nolte A., Gruhn F., Kochba E., Levin Y., Schlensak C., Wendel H.P., Avci-Adali M. (2018). De novo synthesis of elastin by exogenous delivery of synthetic modified mRNA into skin and elastin-deficient cells. Mol. Ther. Nucleic Acids.

[41] Qa'aty N., Wang Y., Wang A., Mao S., Vincent M., Husain M., Hinek A. (2015). The antidiabetic hormone glucagon-like peptide-1 induces formation of new elastic fibers in human cardiac fibroblasts after cross-activation of IGF-IR. Endocrinology.

[42] Mithieux S.M., Aghaei-Ghareh-Bolagh B., Yan L., Kuppan K.V., Wang Y., Garces-Suarez F., Li Z., Maitz P.K., Carter E.A., Limantoro C., Chrzanowski W., Cookson D., Riboldi-Tunnicliffe A., Baldock C., Ohgo K., Kumashiro K.K., Edwards G., Weiss A.S. (2018). Tropoelastin implants that accelerate wound repair. Adv. Healthcare Mater..

[43] Xie H., Lucchesi L., Zheng B., Ladich E., Pineda T., Merten R., Gregory C., Rutten M., Gregory K. (2017). Treatment of burn and surgical wounds with recombinant human tropoelastin produces new elastin fibers in scars. J. Burn Care Res..

[44] Halabi C.M., Mecham R.P., Mecham R.P. (2018). Methods Cell Biol.

[45] Partridge S.M., Davis H.F. (1955). The chemistry of connective tissues. 3. Composition of the soluble proteins derived from elastin. Biochem. J..

[46] Leach J.B., Wolinsky J.B., Stone P.J., Wong J.Y. (2005). Crosslinked α-elastin biomaterials: towards a processable elastin mimetic scaffold. Acta Biomater..

[47] de Vries H.J.C., Middelkoop E., Mekkes J.R., Dutrieux R.P., Wildevuur C.H.R., Westerhof W. (1994). Dermal regeneration in native non-cross-linked collagen sponges with different extracellular matrix molecules. Wound Repair Regen..

[48] Hornebeck W., Tixier J.M., Robert L. (1986). Inducible adhesion of mesenchymal cells to elastic fibers: elastonectin. Proc. Natl. Acad. Sci..

[49] Robert L. (2010). The saga of κ-elastin or the promotion of elastin degradation products from “garbage” to receptor agonists and pharmacologically active principles. Connect. Tissue Res..

[50] Ménasche M., Jacob M.P., Godeau G., Robert A.M., Robert L. (1981). Pharmacological studies on elastin peptides (kappa-elastin). Blood clearance, percutaneous penetration and tissue distribution. Pathol. Biol..

[51] Daamen W.F., van Moerkerk H.T.B., Hafmans T., Buttafoco L., Poot A.A., Veerkamp J.H., van Kuppevelt T.H. (2003). Preparation and evaluation of molecularly-defined collagen–elastin–glycosaminoglycan scaffolds for tissue engineering. Biomaterials.

[52] Masci V.L., Taddei A.R., Gambellini G., Giorgi F., Fausto A.M. (2016). Ultrastructural investigation on fibroblast interaction with collagen scaffold. J. Biomed. Mater. Res..

[53] Walraven M., Akershoek J.J., Beelen R.H.J., Ulrich M.M.W. (2017). In vitro cultured fetal fibroblasts have myofibroblast-associated characteristics and produce a fibrotic-like environment upon stimulation with TGF-β1: is there a thin line between fetal scarless healing and fibrosis?. Arch. Dermatol. Res..

[54] Moulin V., Tam B.Y.Y., Castilloux G., Auger F.A., O'Connor-McCourt M.D., Philip A., Germain L. (2001). Fetal and adult human skin fibroblasts display intrinsic differences in contractile capacity. J. Cell. Physiol..

[55] Kutluoğlu G.C., Vlig M., Elgersma A., Boekema B.K.H.L., Daamen W.F., Doberenz C., Manikowski D. (2025). Comparison of dermal and eschar fibroblasts in full skin equivalents. Wound Repair Regen..

[56] Das P., Manna S., Roy S., Nandi S.K., Basak P. (2023). Polymeric biomaterials-based tissue engineering for wound healing: a systemic review. Burns & Trauma.

[57] Wu X., Zhu H., Xu Y., Kong B., Tan Q. (2023). Chronic wounds: pathological characteristics and their stem cell-based therapies. Eng. Regen..

[58] Huelsboemer L., Knoedler L., Kochen A., Yu C.T., Hosseini H., Hollmann K.S., Choi A.E., Stögner V.A., Knoedler S., Hsia H.C., Pomahac B., Kauke-Navarro M. (2024). Cellular therapeutics and immunotherapies in wound healing – on the pulse of time?. Military Medical Research.

[59] Zhang M., Zhang C., Li Z., Fu X., Huang S. (2022). Advances in 3D skin bioprinting for wound healing and disease modeling. Regen. Biomater..

[60] Mahajan N., Soker S., Murphy S.V. (2024). Regenerative medicine approaches for skin wound healing: from allografts to engineered skin substitutes. Current Transplantation Reports.

[61] Negut I., Dorcioman G., Grumezescu V. (2020). Scaffolds for wound healing applications. Polymers.

[62] Daamen W.F., Nillesen S.T., Wismans R., Reinhardt D., Hafmans T., Veerkamp J.H., van Kuppevelt T.H. (2006). Depots of solubilised elastin promote the formation of blood vessels and elastic fibres in rat. J. Contr. Release.

[63] Schenke-Layland K. (2008). Non-invasive multiphoton imaging of extracellular matrix structures. J. Biophot..

[64] Gade P.S., Robertson A.M., Chuang C.-Y. (2019). Multiphoton imaging of collagen, elastin, and calcification in intact soft-tissue samples. Current Protocols in Cytometry.

[65] König K., Schenke-Layland K., Riemann I., Stock U.A. (2005). Multiphoton autofluorescence imaging of intratissue elastic fibers. Biomaterials.

[66] Thorling C.A., Dancik Y., Hupple C., Medley G., Liu X., Zvyagin A.V., Robertson T., Burczynski F.J., Roberts M.S. (2011). Multiphoton microscopy and fluorescence lifetime imaging provide a novel method in studying drug distribution and metabolism in the rat liver in vivo. J. Biomed. Opt..

[67] Jones J.D., Ramser H.E., Woessner A.E., Quinn K.P. (2018). In vivo multiphoton microscopy detects longitudinal metabolic changes associated with delayed skin wound healing. Commun. Biol..

[68] Krymchenko R., Coşar Kutluoğlu G., van Hout N., Manikowski D., Doberenz C., van Kuppevelt T.H., Daamen W.F. (2024). Elastogenesis in focus: navigating elastic fibers synthesis for advanced dermal biomaterial formulation. Adv. Healthcare Mater..

[69] Halm M., Schenke-Layland K., Jaspers S., Wenck H., Fischer F. (2016). Visualizing tropoelastin in a long-term human elastic fibre cell culture model. Sci. Rep..

[70] Sullivan T.P., Eaglstein W.H., Davis S.C., Mertz P. (2001). The pig as a model for human wound healing. Wound Repair Regen..

[71] Meyerholz D.K., Burrough E.R., Kirchhof N., Anderson D.J., Helke K.L. (2024). Swine models in translational research and medicine. Vet. Pathol..

[72] Nair M., Johal R.K., Hamaia S.W., Best S.M., Cameron R.E. (2020). Tunable bioactivity and mechanics of collagen-based tissue engineering constructs: a comparison of EDC-NHS, genipin and TG2 crosslinkers. Biomaterials.

[73] Sapuła P., Bialik-Wąs K., Malarz K. (2023). Are natural compounds a promising alternative to synthetic cross-linking agents in the preparation of hydrogels?. Pharmaceutics.

