# Supplementary material for: Collagen-elastin dermal scaffolds enhance tissue regeneration and reduce scarring in preclinical models
Source: Mater Today Bio. 2025 Aug 25;34:102239. doi: 10.1016/j.mtbio.2025.102239 (PMC12408406; doi:10.1016/j.mtbio.2025.102239)
Supplement: Multimedia component 1 [file mmc1.docx]

**Supporting information**

**Collagen-elastin dermal scaffolds enhance tissue regeneration and reduce scarring in preclinical models**

Roman Krymchenko^a^, Nancy Avila-Martinez^a^, Merel Gansevoort^a^, Gert-Jan Bakker^a^, Madalena L.N.P. Gomes^b,c,d,e^, Marcel Vlig^b^, Elly M. M. Versteeg^a^, Bouke K. H. L. Boekema^b,d,e^, Toin H. van Kuppevelt^a^, Willeke F. Daamen^a,*^

^a^ Radboud university medical center, Research Institute for Medical Innovation, Department of Medical BioSciences, Geert Grooteplein 28, 6525 GA, Nijmegen, The Netherlands

^b^ Alliance of Dutch Burn Care, Burn Research Lab, Zeestraat 29, 1941 AJ, Beverwijk, The Netherlands

^c^ Department of Pathology, Amsterdam University Medical Center (AUMC), Meibergdreef 9, 1105 AZ, Amsterdam, The Netherlands

^d^ Tissue Function and Regeneration, Amsterdam Movement Sciences Research Institute, Meibergdreef 9, 1105 AZ, Amsterdam, The Netherlands

^e^ Department of Plastic, Reconstructive and Hand Surgery, AUMC, location VUmc, De Boelelaan 1117, 1081 HV, Amsterdam, The Netherlands

*Corresponding author:

**Willeke F. Daamen** - Radboud university medical center, Research Institute for Medical Innovation, Department of Medical BioSciences, Nijmegen, The Netherlands. Email: [Willeke.Daamen@radboudumc.nl](mailto:Willeke.Daamen@radboudumc.nl)


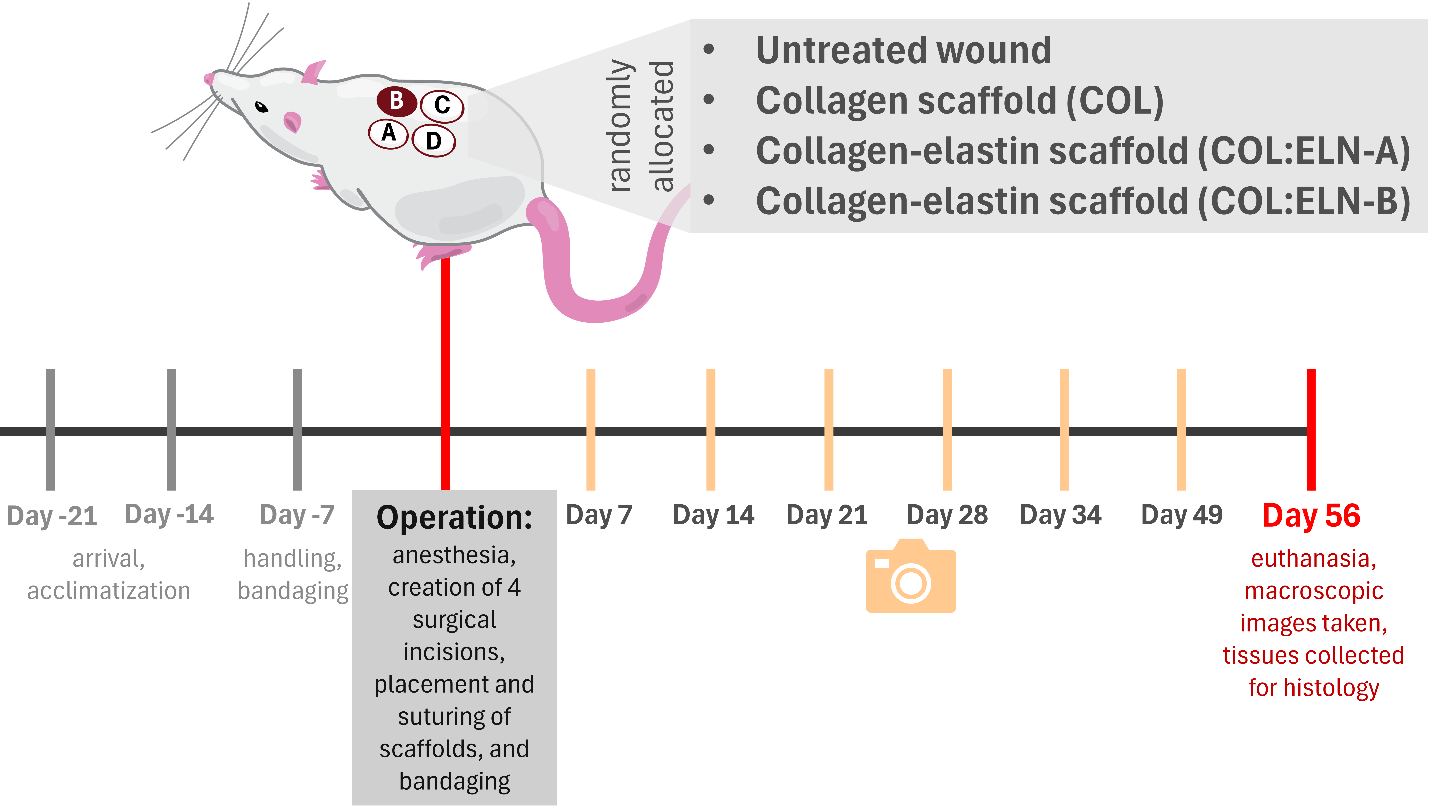


**Figure S1**. Two to three weeks prior to surgery, the rats were delivered to the animal facility and acclimatized. One week after arrival, handling and training were initiated to prepare the animals for the procedures. On the day of surgery, the animals were anesthetized, and four full-thickness wounds were created on the dorsal region (A, B, C, D). Three different scaffolds (COL, COL:ELN-A and COL:ELN-B) were sutured into the wounds in a randomized order. Untreated wounds (without any biomaterial) and wounds treated with COL served as internal controls. The wounds were then covered with dressings and bandages, which were replaced when needed (1-3 times per week). Digital photographs of the wounds were taken at multiple timepoints over a 7-week period to monitor wound healing progression. On day 56, the animals were euthanized, final digital photographs were taken, and skin tissue samples were collected for further analysis.


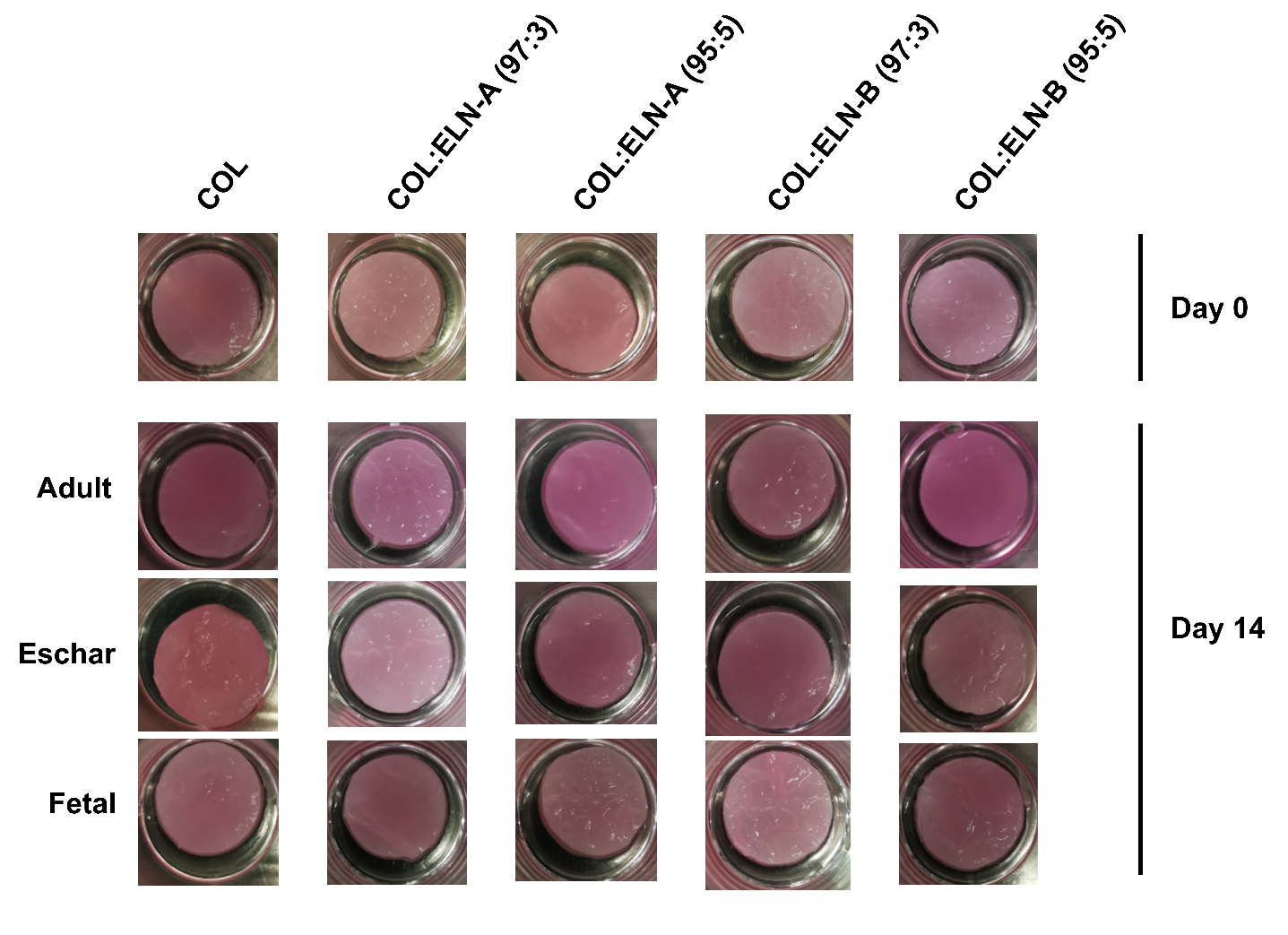


**Figure S2**. Visual appearance of the scaffolds remained the same before seeding the fibroblasts (day 0) and at the end of the cell culture experiment (day 14). COL = type I collagen scaffold, COL:ELN-A = type I collagen scaffold supplemented with ELN-A, COL:ELN-B = type I collagen scaffold supplemented with ELN-B.


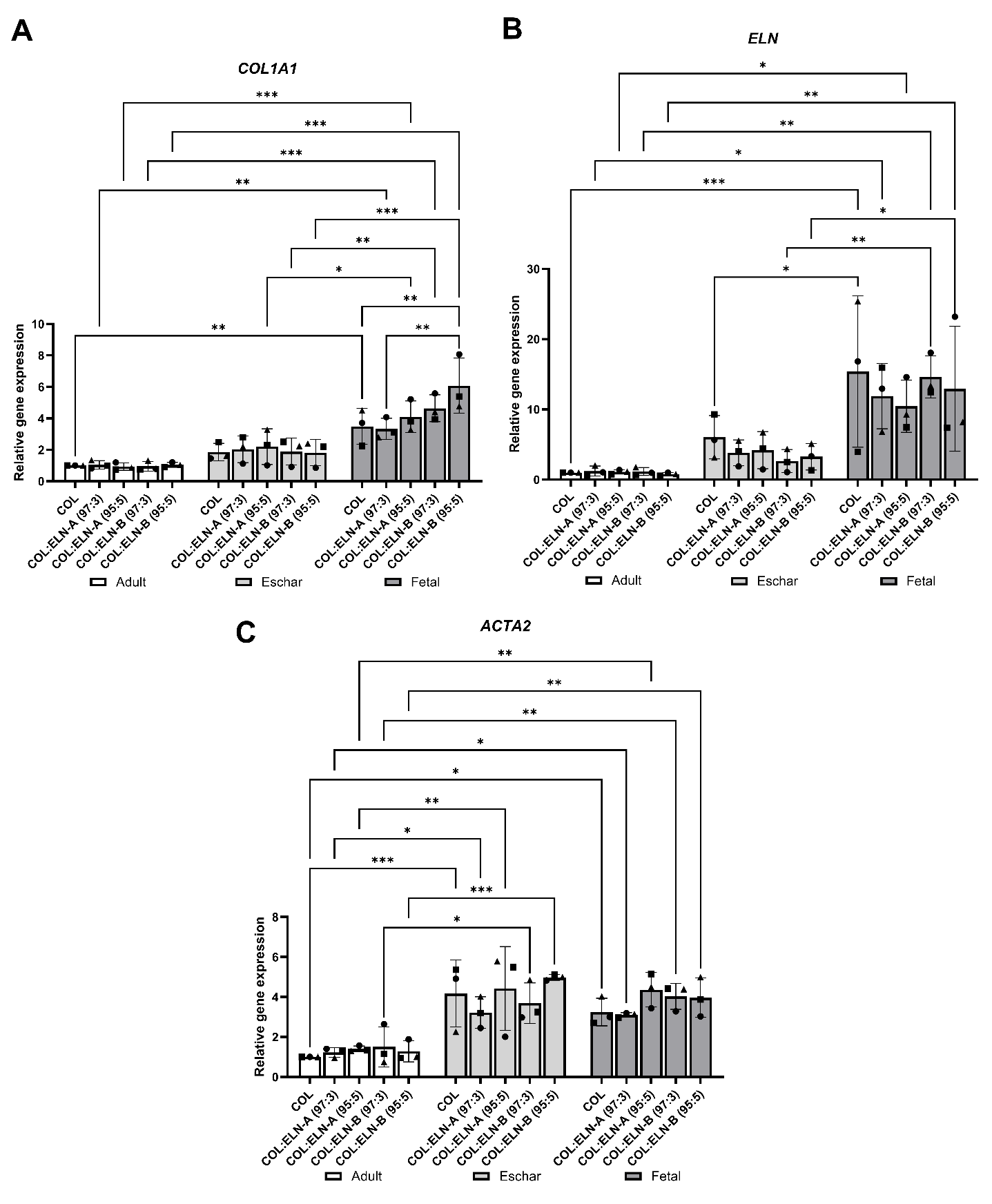


**Figure S3**. Comparative gene expression levels of A) *COL1A1*, B) *ELN*, and C) *ACTA2* across three fibroblast types, normalized to the mean expression of adult fibroblasts cultured on COL scaffolds (day 14, n=3). Individual donors are represented by different symbols as ● donor 1/4/7, ■ donor 2/5/8, and ▲ donor 3/6/9. Differences in gene expression levels between scaffold types and fibroblast types were tested using two-way ANOVA, followed by Tukey’s multiple comparisons test (α = 0.05). Error bars represent standard deviation; * p<0.05, ** p<0.01, *** p<0.001. COL = type I collagen scaffold, COL:ELN-A = type I collagen scaffold supplemented with ELN-A, COL:ELN-B = type I collagen scaffold supplemented with ELN-B, COL1A1 = alpha-1 type I collagen, ELN = elastin, ACTA2 = smooth muscle actin alpha 2.


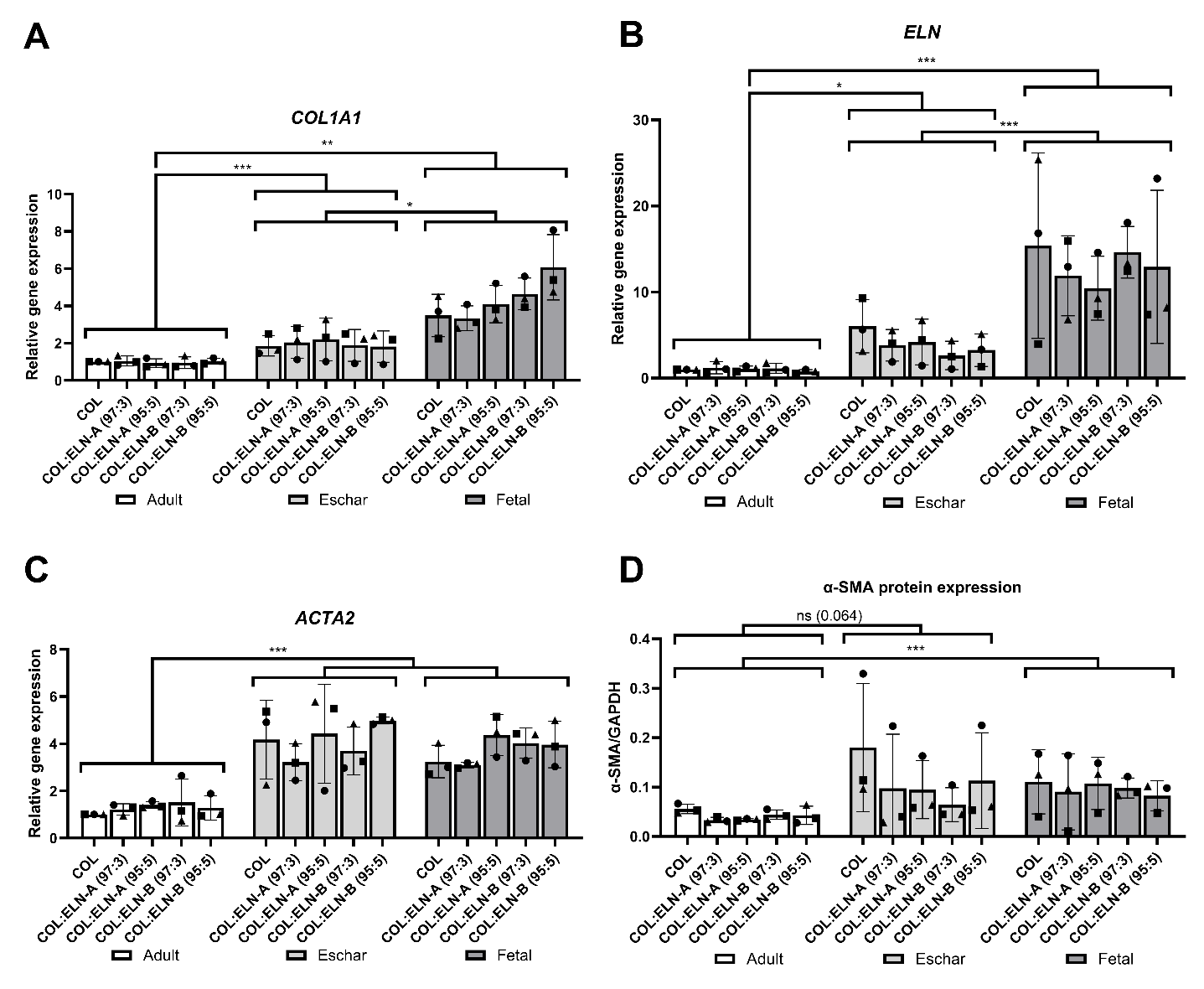


**Figure S4**. Comparative gene expression levels of A) *COL1A1*, B) *ELN*, and C) *ACTA2* across three fibroblast types, normalized to the mean expression of adult fibroblasts cultured on COL scaffolds (day 14, n=3). D) Quantified expression of the α-SMA/GAPDH ratio based on intensities of the bands on Western blot (day 14, n=3). Individual donors are represented by different symbols as ● donor 1/4/7, ■ donor 2/5/8, and ▲ donor 3/6/9. Differences in gene expression levels between fibroblast groups were tested using Brown-Forsythe and Welch ANOVA, followed by Dunnett's T3 multiple comparisons test (α = 0.05). Error bars represent standard deviation; * p<0.05, ** p<0.01, *** p<0.001. COL = type I collagen scaffold, COL:ELN-A = type I collagen scaffold supplemented with ELN-A, COL:ELN-B = type I collagen scaffold supplemented with ELN-B, COL1A1 = alpha-1 type I collagen, ELN = elastin, ACTA2 = smooth muscle actin alpha 2, α-SMA = α-smooth muscle actin.


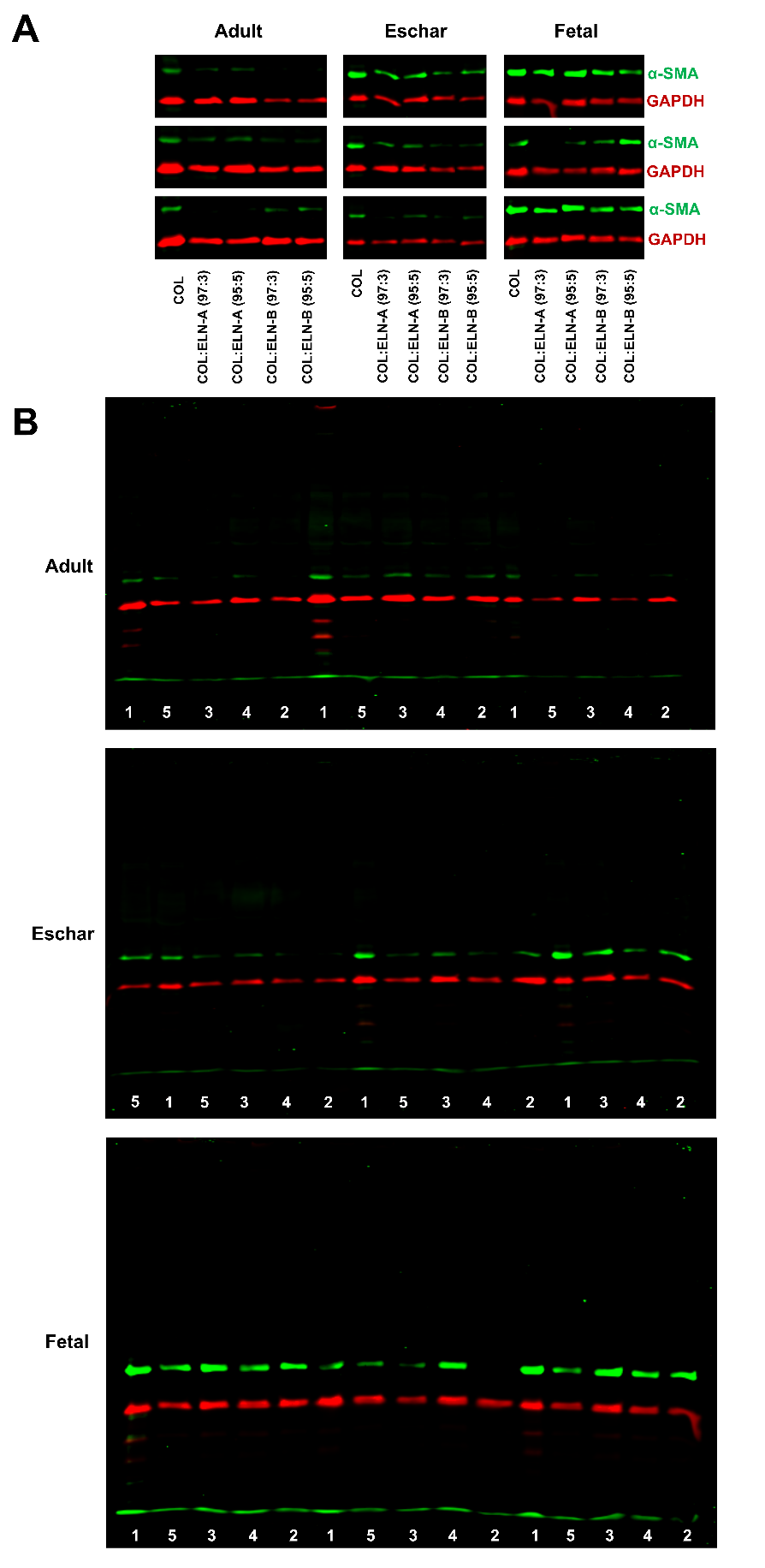


**Figure S5**. A) Cropped and B) uncropped Western blots stained for α-SMA (green, 42 kDa) at day 14. GAPDH expression was used as an internal control (red, 37 kDa). COL = type I collagen scaffold (**1**), COL:ELN-A = type I collagen scaffold supplemented with ELN-A (97:3 – **2**; 95:5 – **3**), COL:ELN-B = type I collagen scaffold supplemented with ELN-B (97:3 – **4**, 95:5 – **5**).


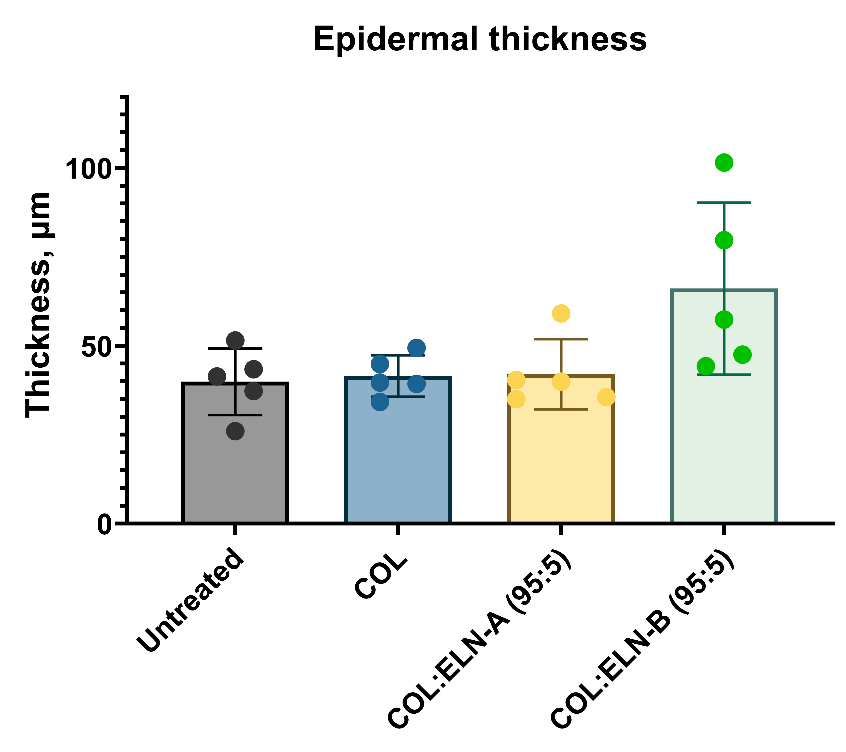


**Figure S6**. Epidermal thickness within four treatment groups. Error bars represent standard deviation.


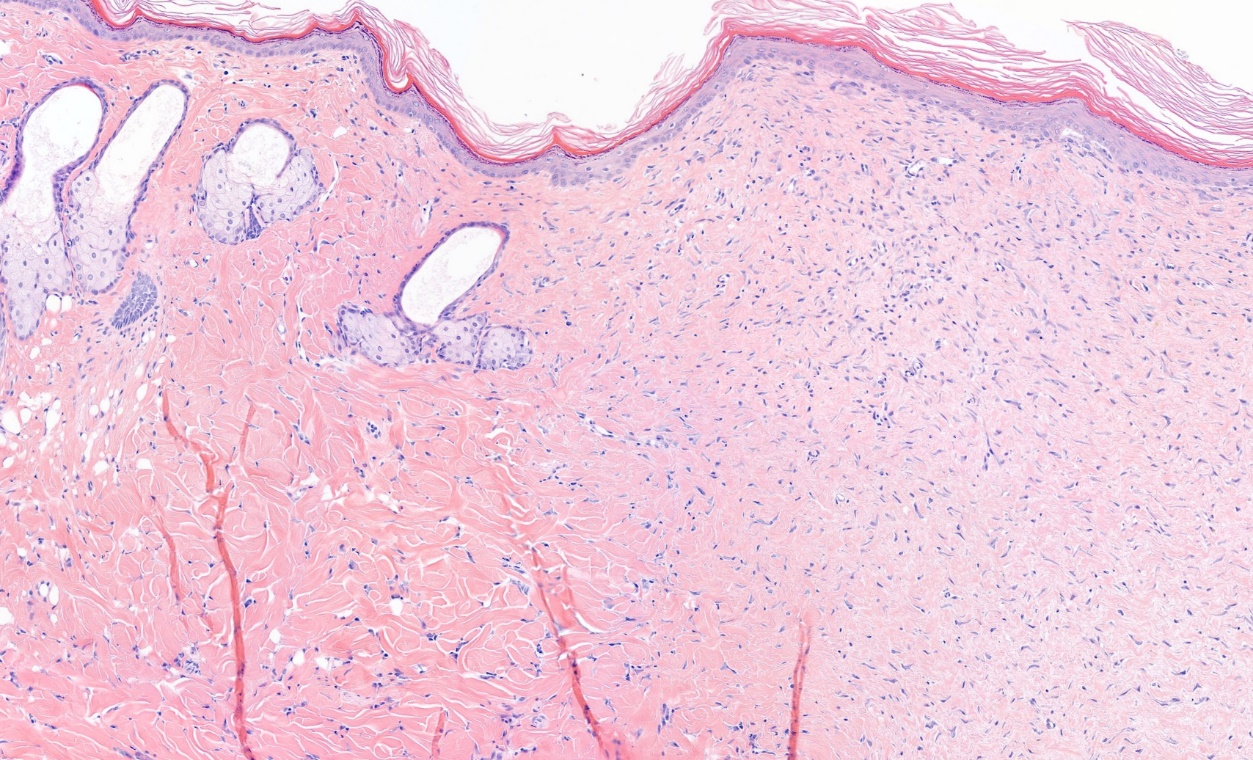


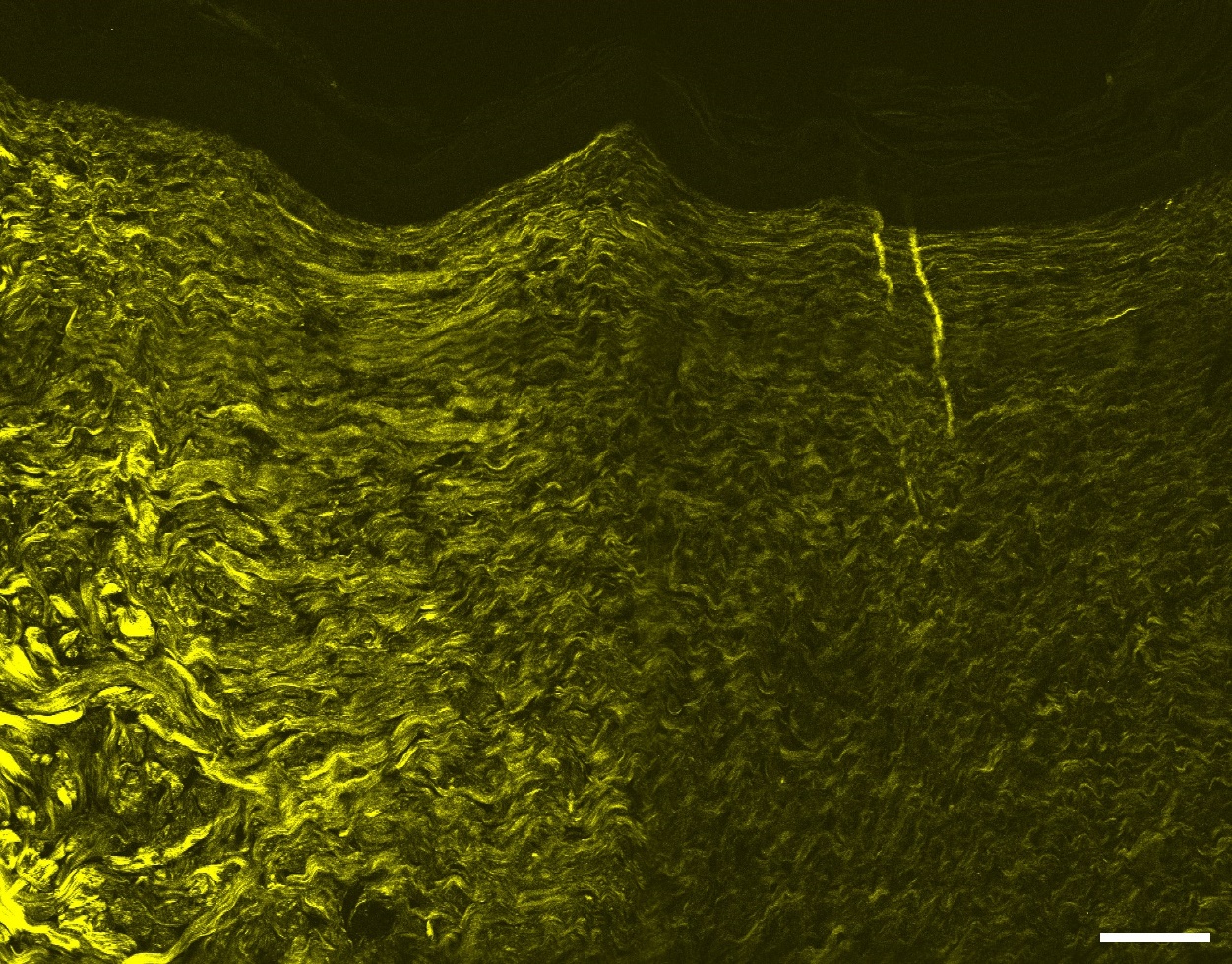


**Figure S7**. Representative H&E (top) and SHG (bottom) images of thick and long collagen fibers in the healthy skin region (white arrow) and thinner and shorter collagen fibers in the wound bed (grey arrow). Scale bars are 100 μm. H&E = hematoxylin and eosin, SHG = second harmonic generation.

|  | **Healthy skin** | **Untreated** | **COL** | **COL:ELN-A (95:5)** | **COL:ELN-B (95:5)** |
| --- | --- | --- | --- | --- | --- |
| **SHG** | 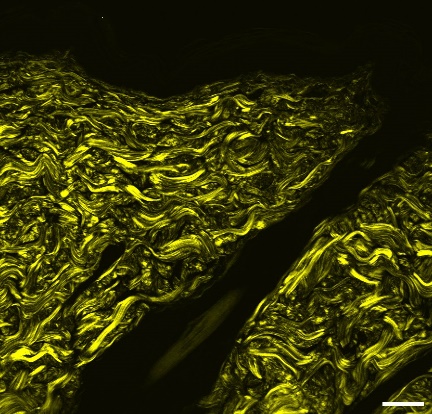 | 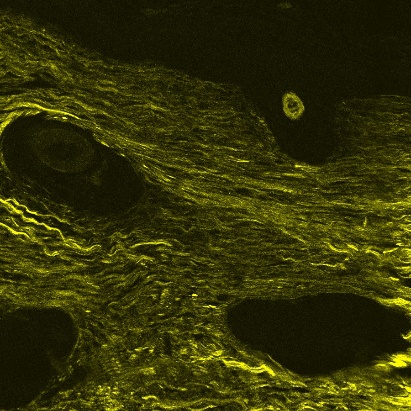 | 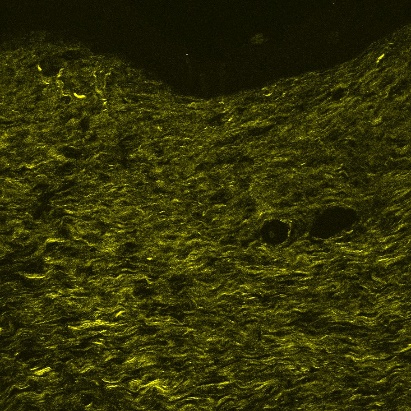 | 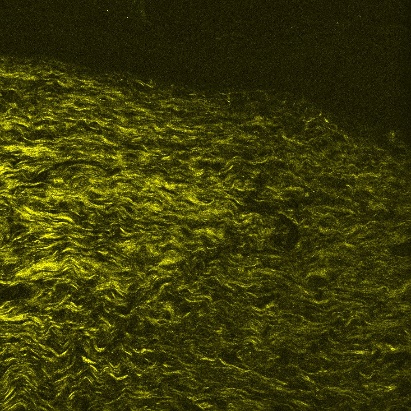 | 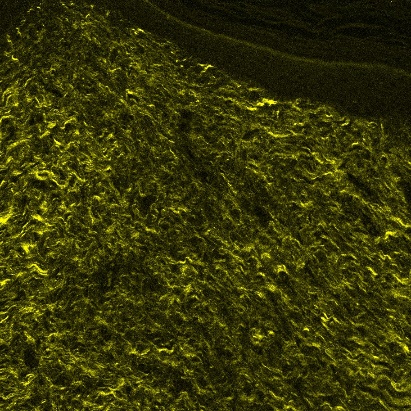 |
| **TPEAF** | 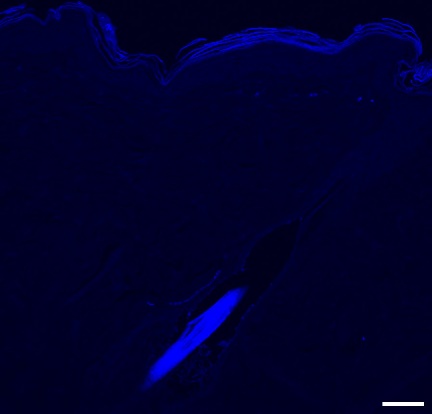 | 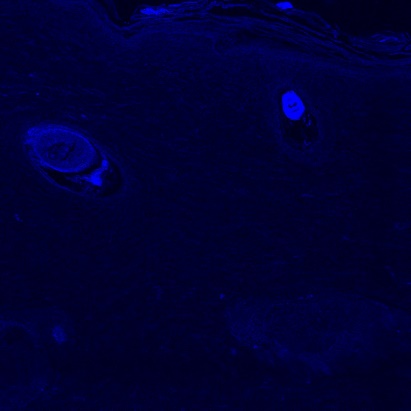 | 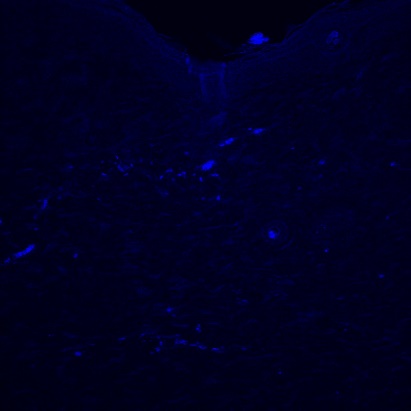 | 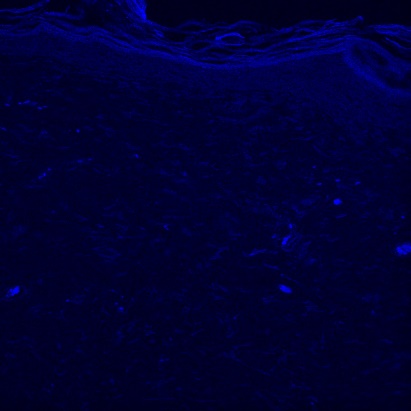 | 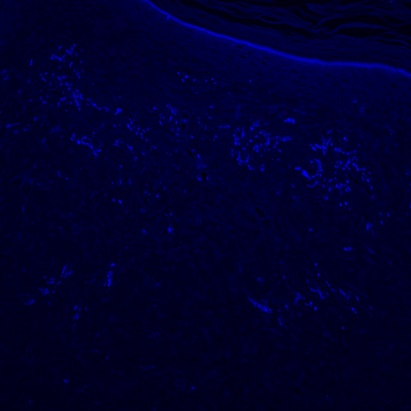 |
| **Merged** | 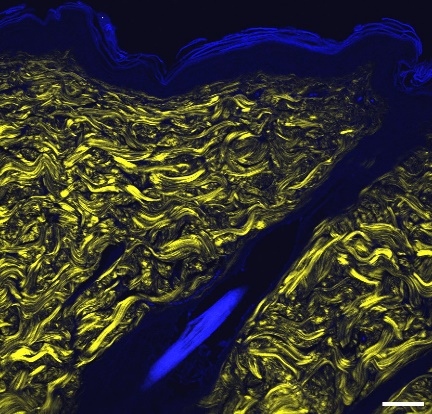 | 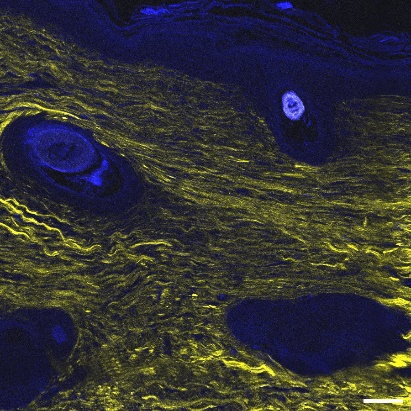 | 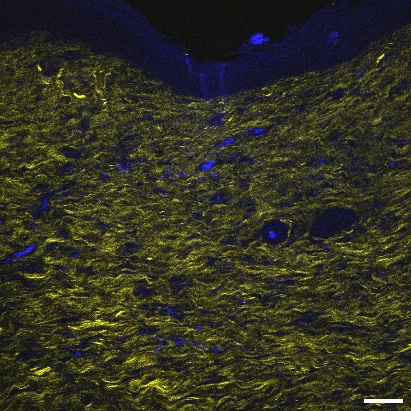 | 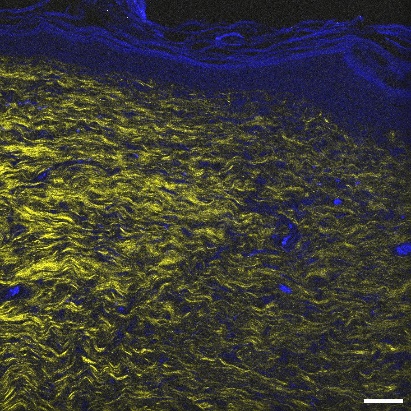 | 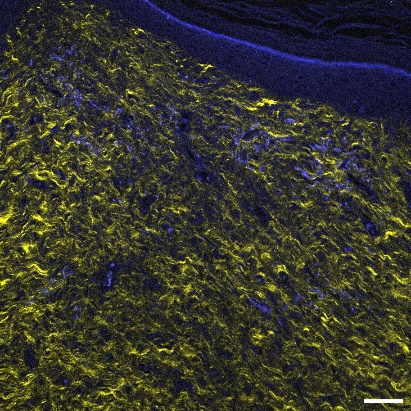 |

**Figure S8**. Multiphoton microscopy images, showing collagen fibers with second harmonic generation (SHG) in yellow and two-photon excited autofluorescence (TFEAF) highlighting endogenous fluorophores in blue. Scale bar is 50 μm. COL = type I collagen scaffold, COL:ELN-A = type I collagen scaffold supplemented with ELN-A, COL:ELN-B = type I collagen scaffold supplemented with ELN-B.

**Table S1. RT-qPCR primer sequences.**

| **Gene** | **Description** | **Forward sequence 5′3′** | **Reverse sequence 5′3′** |
| --- | --- | --- | --- |
| ACTA2 | smooth muscle actin alpha 2 | CCGACCGAATGCAGAAGGA | ACAGAGTATTTGCGCTCCGAA |
| COL1A1 | alpha-1 type I collagen | GAGAGCATGACCGATGGATT | CGCTGTTCTTGCAGTGGTAG |
| ELN | elastin | GGTTGTGTCACCAGAAGCAGCT | CCGTAAGTAGGAATGCCTCCAAC |
| YWHAZ | zeta polypeptide tyrosine 3-monooxygenase/tryptophan 5-monooxygenase activation protein | CATCTTGGAGGGTCGTCTCA | ACTTTGCTCTCTGCTTGTGAA |
| GAPDH | glyceraldehyde 3-phosphate dehydrogenase | TGGGTGTGAACCATGAGAAG | TGGGTGTGAACCATGAGAAG |

**Table S2**. Total RNA ng/µl in fibroblasts seeded on scaffolds after 14 days of culturing.

| **Fibroblasts** | **COL** | **COL:ELN-A (97:3)** | **COL:ELN-A (95:5)** | **COL:ELN-B (97:3)** | **COL:ELN-B (95:5)** |
| --- | --- | --- | --- | --- | --- |
| **Adult** | 110.5 ± 16.5 | 94.1 ± 33.2 | 70.3 ± 27.0 | 87.7 ± 34.0 | 107.9 ± 35.5 |
| **Eschar** | 85.1 ± 25.1 | 123.2 ± 61.2 | 106.0 ± 40.2 | 142.5 ± 49.2 | 136.3 ± 52.3 |
| **Fetal** | 210.3 ± 48.0 | 250.2 ± 54.8 | 236.1 ± 37.5 | 219.7 ± 44.4 | 247.3 ± 48.7 |

N=3, mean ± SD.

**Table S3**. The scaffold size (in cm²) was measured from digital photos of the scaffolds using ImageJ/Fiji.

| **Fibroblasts** | **Timepoint** | **COL** | **COL:ELN-A (97:3)** | **COL:ELN-A (95:5)** | **COL:ELN-B (97:3)** | **COL:ELN-B (95:5)** |
| --- | --- | --- | --- | --- | --- | --- |
| **Adult** | **Day 0** | 1.16 ± 0.03 | 1.14 ± 0.03 | 1.14 ± 0.05 | 1.10 ± 0.04 | 1.20 ± 0.06 |
|  | **Day 3** | 1.18 ± 0.02 | 1.12 ± 0.00 | 1.15 ± 0.02 | 1.14 ± 0.03 | 1.21 ± 0.04 |
|  | **Day 7** | 1.16 ± 0.04 | 1.17 ± 0.02 | 1.17 ± 0.03 | 1.15 ± 0.04 | 1.21 ± 0.05 |
|  | **Day 11** | 1.20 ± 0.04 | 1.14 ± 0.02 | 1.19 ± 0.04 | 1.14 ± 0.03 | 1.24 ± 0.06 |
|  | **Day 14** | 1.16 ± 0.03 | 1.13 ± 0.01 | 1.14 ± 0.03 | 1.13 ± 0.03 | 1.19 ± 0.05 |
| **Eschar** | **Day 0** | 1.15 ± 0.10 | 1.16 ± 0.08 | 1.16 ± 0.09 | 1.17 ± 0.07 | 1.17 ± 0.06 |
|  | **Day 3** | 1.20 ± 0.05 | 1.18 ± 0.05 | 1.22 ± 0.01 | 1.16 ± 0.00 | 1.18 ± 0.03 |
|  | **Day 7** | 1.22 ± 0.03 | 1.18 ± 0.05 | 1.16 ± 0.06 | 1.17 ± 0.05 | 1.19 ± 0.06 |
|  | **Day 11** | 1.17 ± 0.02 | 1.18 ± 0.01 | 1.14 ± 0.02 | 1.16 ± 0.03 | 1.18 ± 0.04 |
|  | **Day 14** | 1.16 ± 0.02 | 1.16 ± 0.05 | 1.18 ± 0.02 | 1.15 ± 0.01 | 1.17 ± 0.05 |
| **Fetal** | **Day 0** | 1.18 ± 0.03 | 1.18 ± 0.02 | 1.23 ± 0.06 | 1.18 ± 0.03 | 1.20 ± 0.03 |
|  | **Day 3** | 1.17 ± 0.04 | 1.19 ± 0.03 | 1.22 ± 0.06 | 1.16 ± 0.01 | 1.16 ± 0.01 |
|  | **Day 7** | 1.14 ± 0.01 | 1.18 ± 0.04 | 1.17 ± 0.01 | 1.14 ± 0.03 | 1.20 ± 0.06 |
|  | **Day 11** | 1.17 ± 0.01 | 1.17 ± 0.03 | 1.19 ± 0.03 | 1.16 ± 0.02 | 1.20 ± 0.04 |
|  | **Day 14** | 1.19 ± 0.04 | 1.19 ± 0.05 | 1.17 ± 0.02 | 1.15 ± 0.03 | 1.19 ± 0.07 |

N=3, mean ± SD.
